# Na^+^ Influx Induced by New Antimalarials Causes Rapid Alterations in the Cholesterol Content and Morphology of *Plasmodium falciparum*

**DOI:** 10.1371/journal.ppat.1005647

**Published:** 2016-05-26

**Authors:** Sudipta Das, Suyash Bhatanagar, Joanne M. Morrisey, Thomas M. Daly, James M. Burns, Isabelle Coppens, Akhil B. Vaidya

**Affiliations:** 1 Center for Molecular Parasitology, Department of Microbiology and Immunology, Drexel University College of Medicine, Philadelphia, Pennsylvania, United States of America; 2 Department of Molecular Microbiology and Immunology, Johns Hopkins School of Public Health, Baltimore, Maryland, United States of America; University of Texas Southwestern Medical Center, UNITED STATES

## Abstract

Among the several new antimalarials discovered over the past decade are at least three clinical candidate drugs, each with a distinct chemical structure, that disrupt Na^+^ homeostasis resulting in a rapid increase in intracellular Na+ concentration ([Na^+^]_i_) within the erythrocytic stages of Plasmodium falciparum. At present, events triggered by Na^+^ influx that result in parasite demise are not well-understood. Here we report effects of two such drugs, a pyrazoleamide and a spiroindolone, on intraerythrocytic P. falciparum. Within minutes following the exposure to these drugs, the trophozoite stage parasite, which normally contains little cholesterol, was made permeant by cholesterol-dependent detergents, suggesting it acquired a substantial amount of the lipid. Consistently, the merozoite surface protein 1 and 2 (MSP1 and MSP2), glycosylphosphotidylinositol (GPI)-anchored proteins normally uniformly distributed in the parasite plasma membrane, coalesced into clusters. These alterations were not observed following drug treatment of *P*. *falciparum* parasites adapted to grow in a low [Na^+^] growth medium. Both cholesterol acquisition and MSP1 coalescence were reversible upon the removal of the drugs, implicating an active process of cholesterol exclusion from trophozoites that we hypothesize is inhibited by high [Na^+^]_i_. Electron microscopy of drug-treated trophozoites revealed substantial morphological changes normally seen at the later schizont stage including the appearance of partial inner membrane complexes, dense organelles that resemble “rhoptries” and apparent nuclear division. Together these results suggest that [Na^+^]_i_ disruptor drugs by altering levels of cholesterol in the parasite, dysregulate trophozoite to schizont development and cause parasite demise.

## Author Summary

Malaria remains a major public health challenge in the world, especially with the realization that parasites causing the disease are becoming resistant to currently used antimalarial drugs. There are new antimalarial drugs under development, and among these are 3 clinical candidate drugs that have the propensity to cause sodium leakage into parasites growing inside human red blood cells. We have investigated events that lead to parasite death when sodium concentration inside the parasite increases. Our findings suggest that the drug-treated parasite rapidly acquires cholesterol and clusters containing lipid-embedded proteins MSP1 and MSP2 form within the plasma membrane. Because these changes are reversible when the drugs are removed, we suggest that there is an active process that keeps cholesterol out of the parasite. We also observed massive morphological changes resembling premature steps of parasite division following drug treatment. The changes we describe appear to be a direct consequence of increased sodium level in the parasites. We hypothesize that sodium influx constitutes a normal signaling process in malaria parasites and that the new antimalarial drugs initiate this process prematurely, which results in the death of the parasites.

## Introduction

Billions of people living in regions endemic for malaria are confronted with the looming threat of *Plasmodium falciparum* parasites resistant to currently effective artemisinin combination therapies [1]. From an evolutionary point of view, emergence of resistant parasites has to be anticipated, especially in light of the fact that the drug pressure is being applied on a vast population of parasites, whose transmission requires obligatory sexual reproduction favoring recombinatorial selection of beneficial drug resistance alleles. Thus for a foreseeable future, efforts to control and eliminate malaria will require a robust pipeline of antimalarial drugs under development. Over the past decade, efforts by academic and industrial investigators have begun to prime this pipeline with new chemical entities with potent antimalarial activity [2]. Understanding the mechanism by which these new compounds cause the demise of malaria parasites would reveal vulnerable aspects of parasite physiology, which in turn could identify other new potential drug targets for further investigations.

**Fig 1:**
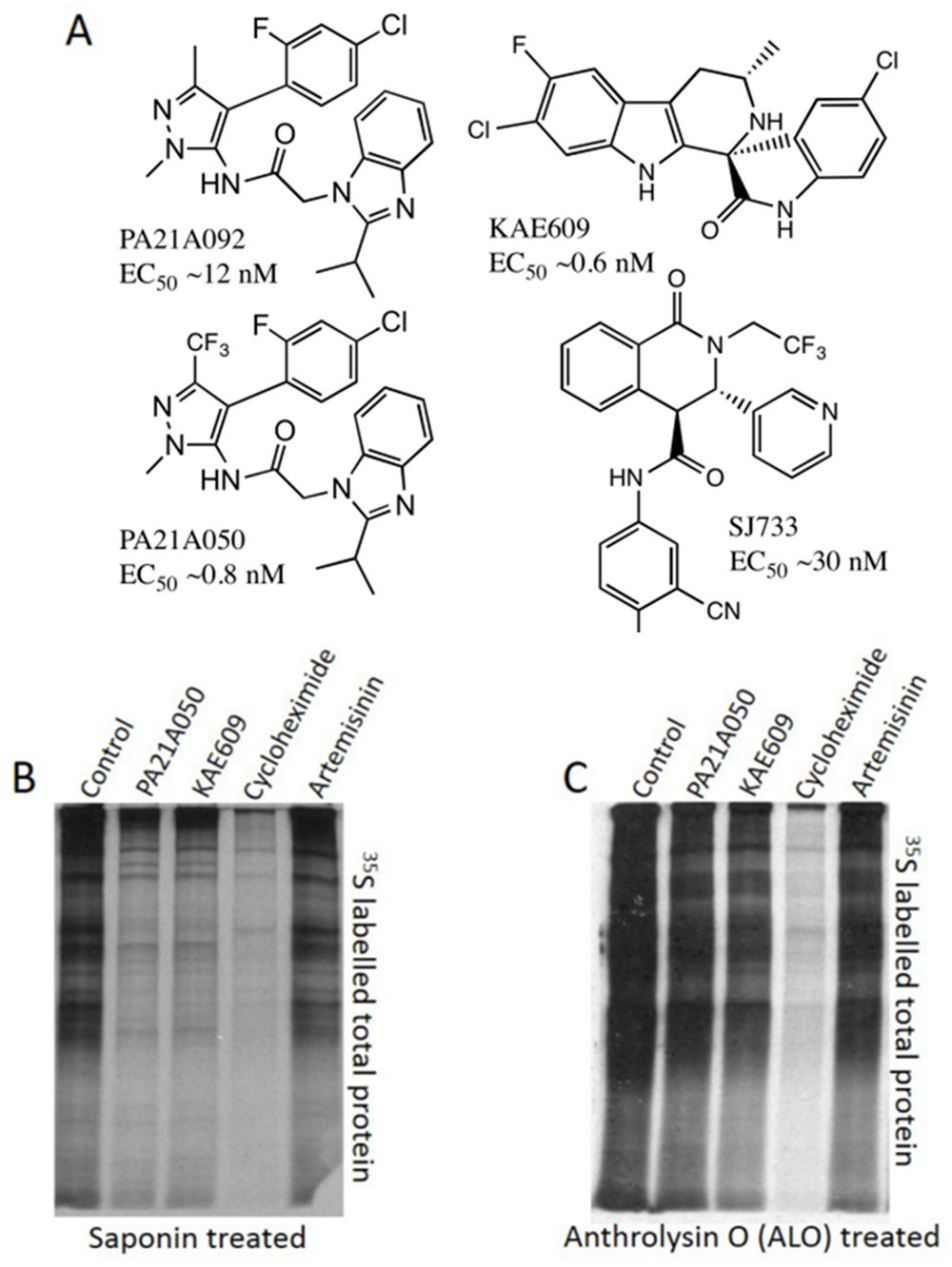
Structures of [Na^+^]_i_ disruptor compounds and their effect on protein synthesis. (A) Structures of compounds discussed in the text and their EC_50_ values. (B) Pyrazoleamide and spiroindolones do not inhibit parasite protein synthesis. 32–34 h PMI (post merozoite invasion) trophozoite stage *P*. *falciparum* 3D7 parasites were exposed to the vehicle (Control) or to 10x EC_50_ (10 nM) of PA21A050, KAE609, cycloheximide (2000 nM), or artemisinin (100 nM) for 2h in a ^35^S-methionine/^35^S-cysteine containing medium. Autoradiograph of labeled total proteins in saponin-freed (B) and anthrolysin O (ALO)-freed parasites (C) are shown. Isolated parasites with saponin led to detection of a highly reduced amount of proteins in PA21A050 and KAE609 treated parasites but not when parasites were freed by ALO.

Three new antimalarials designated as clinical candidates, each with a distinct chemical structure, appear to share a common mode of action. These antimalarial drugs—belonging to the spiroindolone [3,4], pyrazoleamide [5] and dihydroisoquinolone (DHIQ) [6] chemical classes (see Fig 1A for structures) were all shown to induce a rapid influx of Na^+^ into isolated trophozoite stages of *P*. *falciparum*. An investigation of about 400 antimalarial compounds included in the “Malaria Box” supplied by the Medicines for Malaria Venture (MMV) revealed 28 compounds belonging to 16 distinct chemical classes that also caused rapid Na^+^ influx into isolated *P*. *falciparum* trophozoites [7]. Resistance to several of the compounds causing Na^+^ influx was found to be associated with mutations within a P-type cation ATPase, PfATP4 [4,5,6], a plasma membrane transporter initially annotated as a non-SERCA Ca^2+^ ATPase pump [8], but now revealed to have characteristics of a Na^+^ pump [3]. The existence of resistance-associated mutations in PfATP4 has been interpreted as indicating that the large number of chemical classes causing Na^+^ influx into isolated trophozoites are all direct inhibitors of PfATP4, and that it is the inhibition of the Na^+^ efflux function of PfATP4 that leads to the rapid increase in [Na^+^]_i_. While this appears to be a reasonable interpretation, there is no direct evidence at this time to show that PfATP4 is indeed a Na^+^ pump, or that various antimalarials causing [Na^+^]i disruption directly inhibit its Na^+^ pumping activity. Genetic investigations of pyrazoleamide-resistant *P*. *falciparum* have revealed potential epistatic interactions between PfATP4 and regulatory molecules such as a Ca^2+^-dependent protein kinase [5]. Thus, it appears possible that Na^+^ homeostasis within the intraerythrocytic stages of *P*. *falciparum* might be regulated by a complex network, and that perturbation of this network by a variety of small molecules could also lead to Na^+^ homeostasis disruption.

Cellular life depends upon maintenance of appropriate intracellular ionic concentrations. All cells expend substantial amount of energy for this purpose. Numerous transporters and their associated regulators manage ionic homeostasis, especially for intracellular [H^+^], [Na^+^], [K^+^] and [Ca^2+^]. This results in concentration gradients of ions across the plasma membranes, which in turn permit rapid changes in intracellular ionic concentrations, the basis for a wide range of signaling cascades affecting cellular physiology. Maintenance of ionic homeostasis is especially challenging to an intracellular eukaryotic parasite such as *Plasmodium*. Intraerythrocytic stages of the parasite are confronted with a changing ionic environment of the erythrocyte cytosol. As the parasite matures, new permeability pathways induced by the parasite result in increased [Na^+^] within the host cell [9]. Hence, Na^+^ pumping by PfATP4 is proposed as a means to maintain physiological levels of [Na^+^]_i_ [3]. Although drug-induced influx of Na^+^ within the parasite is likely to be disruptive to parasite physiology, details of the events triggered by the resultant ionic imbalance that eventually lead to parasite death have not been explored. We report here dramatic alterations in parasite plasma membrane permeability and morphology induced by two chemically distinct [Na^+^]_i_ disruptor drugs. These changes resemble the last stages of schizogony, which leads us to suggest Na^+^ influx as a signaling event that is prematurely induced by these antimalarial drugs.

## Results

### PA21A050 and KAE609 rapidly induce saponin-sensitivity in *P*. *falciparum*

The initial publication on the spiroindolone KAE609 reported rapid protein synthesis inhibition in intraerythrocytic *P*. *falciparum* as a possible reason for the parasite demise (Fig 2 in Rottman et al. [4]). The authors used ^35^S-amino acid incorporation into trichloroacetic acid (TCA) insoluble radioactive material over a 1 h exposure to the drug to assess protein synthesis by control and drug-treated parasites. The TCA precipitation was carried out after the parasites were released by treatment with saponin to eliminate the quenching effect of hemoglobin released from the erythrocytes. When we assessed protein synthesis inhibition by autoradiography of newly synthesized proteins displayed by SDS-polyacrylamide gel electrophoresis (PAGE), we also found a much reduced amount of labeled proteins in saponin-released KAE609 and PA21A050-treated parasites (Fig 1B). However, when parasites were released by a milder treatment using anthrolysin-O (ALO), a cholesterol-dependent cytolysin [10], we observed a minimal reduction of newly synthesized proteins in KAE609- and PA21A050-treated samples, as determined by autoradiography (Fig 1C). We used cycloheximide and artemisinin, as positive and negative controls respectively, and observed the expected protein synthesis inhibition by cycloheximide and minimal inhibition by artemisinin, whether the parasites were freed by saponin or ALO. These results suggested that a short treatment with KAE609 or PA21A050 causes the parasite plasma membrane to become sensitive to saponin detergent, resulting in the leakage of cytosolic proteins, and that these drugs do not induce rapid protein synthesis inhibition.

**Fig 2:**
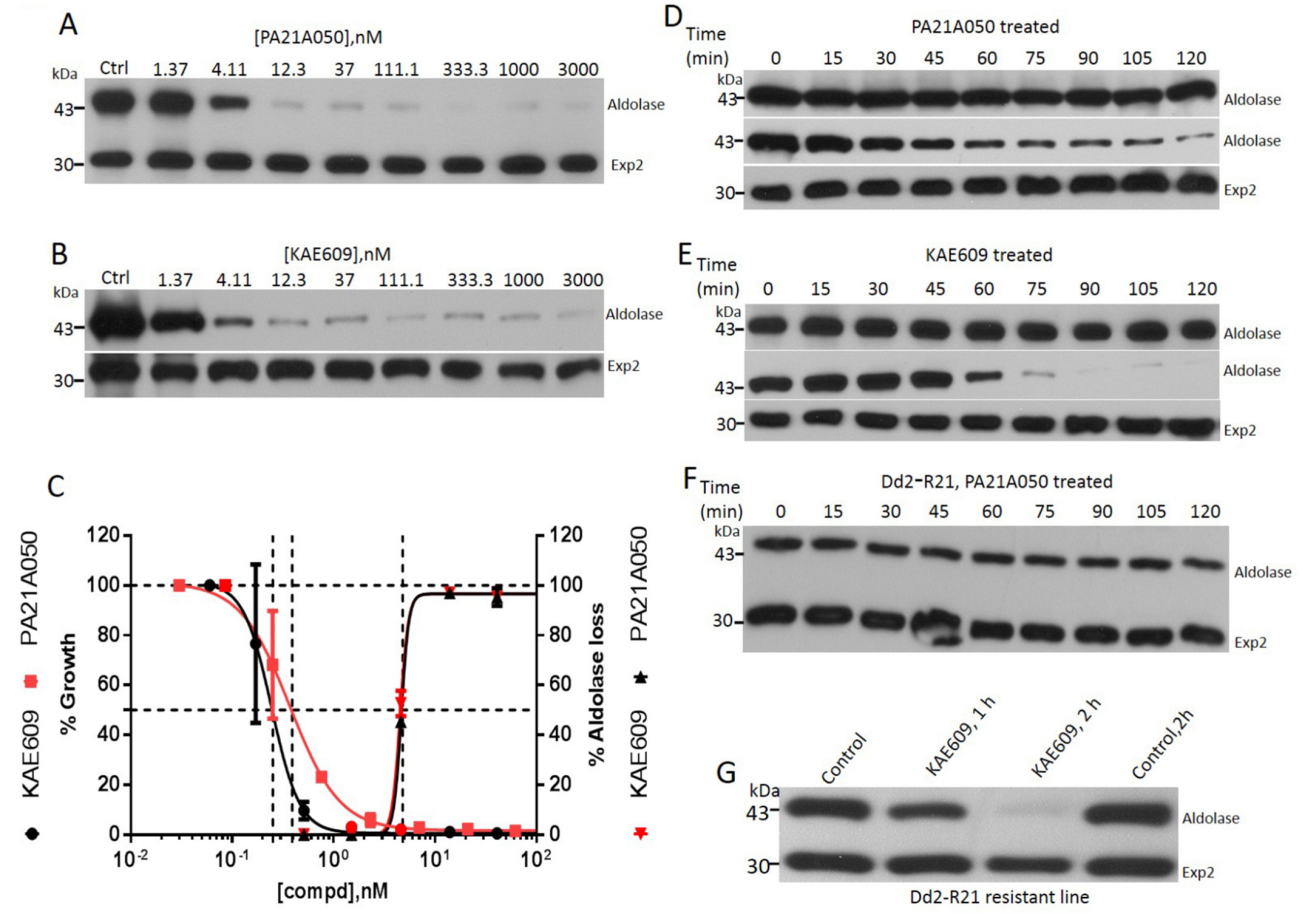
Pyrazoleamide and spiroindolone antimalarials induce saponin sensitivity to the PPM. Trophozoite stage *P*. *falciparum* 3D7 (30–34 h post-infection) were exposed to the vehicle (Ctrl) or the indicated doses of PA21A050 (A) or KAE609 (B) for 2 h, followed by mild saponin treatment to release the parasites and subjected to Western blot analysis using antibodies to aldolase or Exp2. (C) Densitometric measures (using Image J) of the band intensities from the Western blots were plotted as functions of compound concentrations to derive EC_50_ values for aldolase loss in a 2 h exposure. Growth inhibition by PA21A050 and KAE609 was assessed by ^3^H-hypoxanthine incorporation by *P*. *falciparum* in a 48 h assay. Saponin-freed parasites were exposed to 10 nM PA21A050 (D) or KAE609 (E) for the indicated time. Parasite proteins were subjected to SDS-PAGE without further treatment (upper panels in D and E) or after subjecting the parasites to a second saponin treatment (middle and lower panels in D and E). Western blots were probed with antibodies to aldolase or Exp2. Pyrazoleamide resistant line, Dd2-R21 parasitized erythrocytes were exposed to 10 nM PA21A050 (F) and 10 nM KAE609 (G) for the indicated time followed by saponin treatment and Western blotting and probing with antibodies to aldolase or Exp2. Error bars represent the standard deviation (SD) of the measurements. All western blot images are the representative of multiple biological replicates.

Saponin treatment is widely used to free intact parasites from erythrocytes without apparent permeation of the parasite plasma membrane (PPM), and has permitted extensive investigations of transport across the PPM [11–15]. This is likely due to the highly reduced cholesterol content of the PPM compared to the erythrocyte membrane and the parasitophorous vacuolar membrane (PVM) [16–18]. The increased susceptibility of the parasites to permeation by saponin in drug-treated parasites led us to examine this phenomenon in some detail. Both PA21A050 and KAE609 induced saponin sensitivity to the parasites in a dose dependent manner, as judged by the loss of aldolase, a cytosolic protein. Treatment with artemisinin did not cause saponin-induced aldolase leakage (S1B Fig). Exposure to PA21A050 and KAE609 did not affect the level of an integral PVM protein Exp2 (Fig 2A and 2B), or the level of an integral PPM protein PfATP4 (S1A Fig). This indicated that the loss of cytosolic aldolase likely occurred through saponin-induced pores within the PPM of treated parasites rather than by dissolution of the membranes. The effective concentrations for 50% loss of aldolase (EC_50_) were about 10 fold higher in this 2 h assay compared to the EC_50_ observed for parasite growth inhibition carried out for 48 h (Fig 2C). Induction of saponin-sensitivity was also observed when freed parasites were exposed to the drugs. This was observable within 30–60 min of exposure (Fig 2D and 2E). Furthermore, a pyrazoleamide-resistant *P*. *falciparum* line, Dd2-R21 [5], did not develop saponin sensitivity when exposed to PA21A050, thereby showing a clear link between the antimalarial drug action and the acquisition of saponin sensitivity (Fig 2F). The Dd2-R21 line is not cross-resistant to KAE609 [5], and therefore acquired saponin sensitivity when exposed to this drug (Fig 2G). Parasites released from the erythrocytes by exposure to hypotonic buffer did not acquire saponin sensitivity (S2 Fig). Taken together, these data establish that the rapid induction of the saponin sensitivity of the trophozoites is a result of exposure to these drugs.

### The saponin sensitivity is due to rapid accumulation of cholesterol by the trophozoites in response to the drugs

Saponin is a mixture of cholesterol-dependent glycoside detergents similar to digitonin. We found that digitonin also caused aldolase leakage in treated parasites (S1C Fig). These results strongly suggested that exposure to these new antimalarials rapidly leads to cholesterol acquisition by the parasites. We assessed this by using methyl-β-cyclodextrin (MβCD) to extract cholesterol from membranes. MβCD is composed of a cyclic polymer of 7 glucose monomers bearing varying degrees of methylation. Thus, MβCD, while being water soluble, possesses a hydrophobic cavity within the cyclic polymer with the capacity to extract highly hydrophobic cholesterol from lipid bilayers. Measurement of cholesterol content in freed parasites using a biochemical assay (Amplex Red Cholesterol Assay kit; ThermoFisher Scientific) showed >50% reduction in total cholesterol content after the freed parasites were extracted with 5 mM MβCD (S1 Table). Re-incubation of MβCD-extracted (i.e. cholesterol depleted) freed parasites with cholesterol-saturated MβCD resulted in repletion of the total cholesterol content of the sample (S1 Table). Thus, the use of MβCD with or without added cholesterol permits assessment of the involvement of cholesterol in inducing saponin sensitivity following drug treatments. For many years, MβCD extraction has been widely employed for investigating cholesterol dynamics in cell biology [17,19–21].

**Fig 3:**
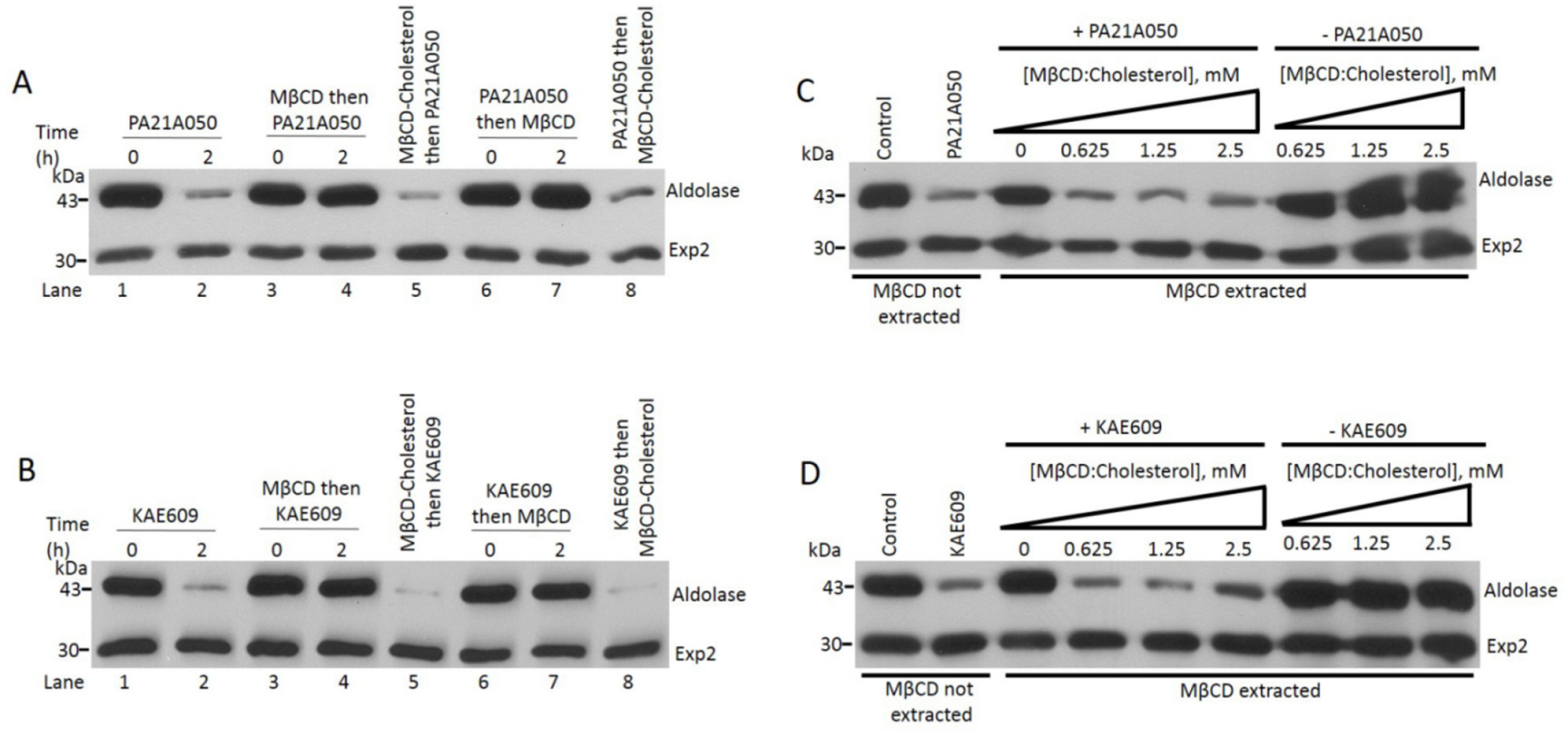
Pyrazoleamide and spiroindolone induced saponin sensitivity is due to cholesterol incorporation into the parasite. (A and B) Cholesterol extraction with MβCD eliminates saponin sensitivity induced by the drugs, whereas cholesterol-loaded MβCD does not. Freed 30–34 h post-infection trophozoites were treated with either PA21A050 (A) or KAE609 (B). Parasites were treated with the drugs (lanes 1 and 2), or were exposed to MβCD to extract cholesterol and then treated with the drugs (lanes 3 and 4). Freed parasites were exposed to MβCD loaded with cholesterol followed by the drug treatment (lanes 5). Freed parasites were first treated with the drugs and then exposed to either MβCD (lanes 6 and 7) or MβCD loaded with cholesterol (lanes 8). After the indicated time, parasites were subjected to a brief exposure to saponin, centrifuged and subjected to SDS-PAGE followed by immunobloting with antibodies to aldolase and Exp2. (C and D) Cholesterol loaded MβCD can donate cholesterol to cholesterol-depleted freed parasites, restoring drug-induced saponin sensitivity. Experiments with PA21A050 treatment (C) and KAE609 treatment (D) are shown. Inclusion of just 0.625 mM cholesterol-loaded MβCD in cholesterol-depleted parasites restored saponin sensitivity to parasites when treated with the drugs. However, in absence of the drugs inclusion of cholesterol-loaded MβCD did not impart saponin sensitivity, suggesting an active process of cholesterol exclusion from the parasite.

Freed parasites were subjected to MβCD extraction, either prior to, or after the treatment with PA21A050, which was then followed by assessment of the saponin-sensitivity of the PPM as revealed by aldolase leakage. Fig 3A shows that cholesterol extraction by MβCD either prior or subsequent to the treatment with PA21A050 prevented saponin-dependent aldolase leakage from the parasite. Similar results were also seen when KAE609 was used to treat parasites (Fig 3B). Thus, ~50% depletion of cholesterol content of freed parasites (S1 Table) appears to eliminate drug-induced saponin sensitivity of the PPM. Pre-loading MβCD with cholesterol (which would prevent its ability to extract cholesterol from the parasites) prior to its use in these experiments resulted in aldolase leakage when exposed to saponin (Fig 3A and 3B, lanes 5 and 8). We also observed that freed parasites extracted with MβCD, while being resistant to saponin-mediated aldolase leakage when exposed to PA21A050 and KAE609, were made sensitive to saponin if co-incubated with MβCD preloaded with cholesterol (Fig 3C and 3D).

Exposure of MβCD extracted freed parasites to cholesterol-loaded MβCD did not result in acquisition of saponin sensitivity when not treated with the compounds (Fig 3C and 3D), suggesting the possibility of an active process that excludes cholesterol from the parasites under normal conditions (discussed further below). Overall these observations provide strong support to the interpretation that treatment with these compounds results in rapid cholesterol incorporation into the parasite including PPM, and that the source of cholesterol is external to the parasite, likely being derived from the PVM, the RBC plasma membrane, or both.

### Rapid cholesterol incorporation by trophozoites requires influx of Na^+^ from an external source

**Fig 4:**
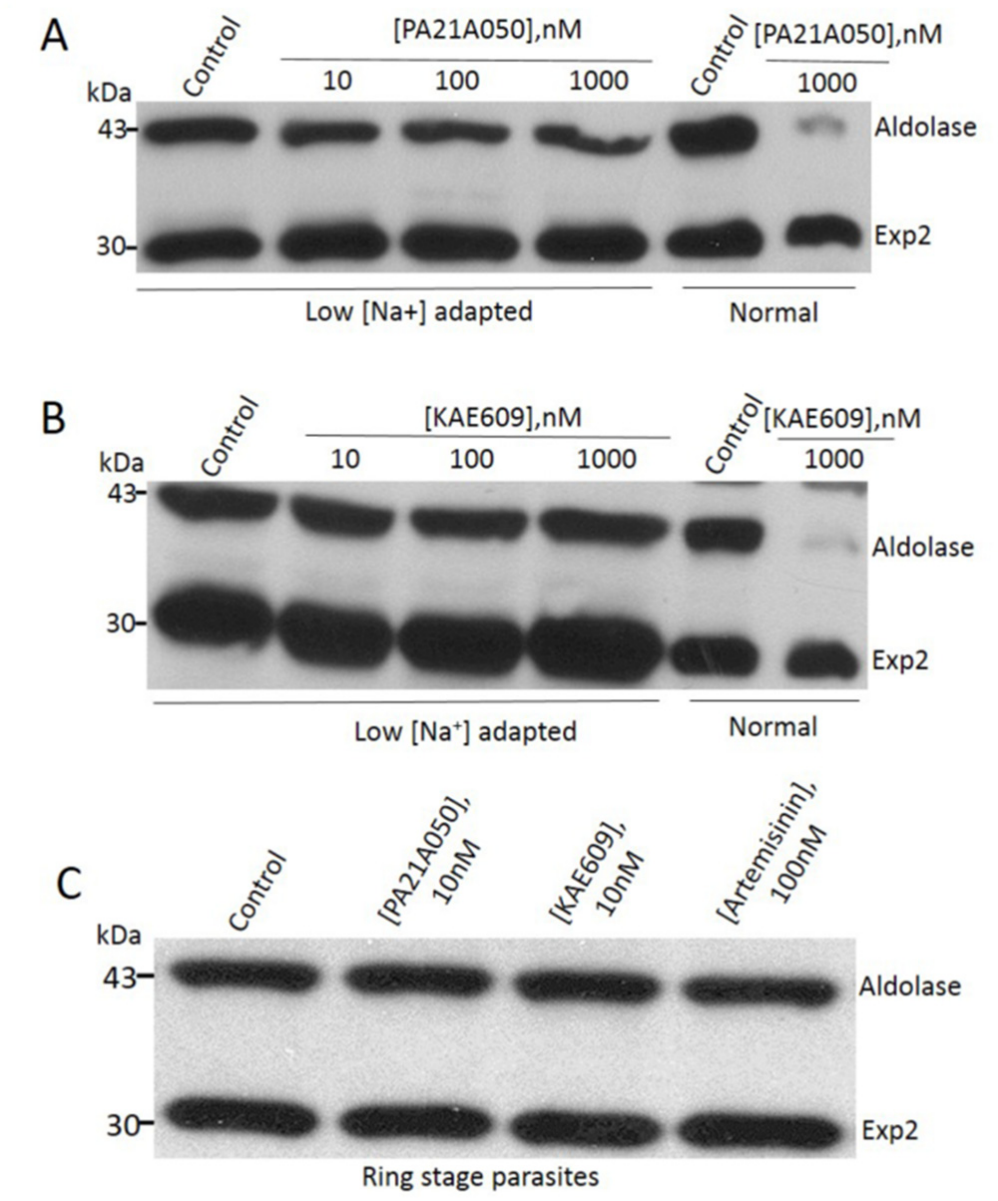
Na^+^ influx induction is required for cholesterol acquisition by the parasite in PA21A050 and KAE609 treated parasites. Trophozoite stage *P*. *falciparum* parasites (30–34 h post-infection), adapted to grow in low [Na^+^] medium, were treated with the indicated concentration of the PA21A050 (A) or KAE609 (B). Cholesterol acquisition was assessed by sensitivity to saponin-mediated loss of aldolase, which was absent in parasites grown in low [Na^+^] medium but apparent in parasites grown in normal medium. (C) Saponin sensitivity was not induced by the compounds in parasites at the ring stage where the erythrocyte cytosol does not yet contain a high [Na^+^] and the parasites are not exposed to the high [Na^+^] of the medium. Western blots were probed with antibodies to aldolase and Exp2. All western blot images are representative of multiple biological replicates.

Cholesterol incorporation into the usually cholesterol-deficient PPM could have significant impact on the functioning of PPM-resident proteins such as transporters. Since the rapid increase in [Na^+^]_i_ in treated parasites (S3 Fig) is proposed to be due to inhibition of a P-type Na^+^ pump, PfATP4, we wondered whether the disruption of Na^+^ homeostasis by these drugs preceded cholesterol acquisition by the parasites, or whether the elevated cholesterol caused inhibition of Na^+^ pumping by PfATP4, leading to an increased [Na^+^]_i_. To answer this question, we took advantage of *P*. *falciparum* parasites adapted to grow in a low [Na^+^] growth medium [22]. Parasites growing in low [Na^+^] medium would be exposed to low [Na^+^] within the host cell cytosol, since Na^+^ concentration within the host cell would be expected to equilibrate with the growth medium. Treatment of low [Na^+^] adapted parasites with the drugs did not result in induction of saponin sensitivity of the PPM (Fig 4A and 4B). These results suggest that cholesterol acquisition in treated parasites is a consequence of Na^+^ homeostasis disruption. We observed that drug treatment of the ring-stage parasites did not result in the acquisition of saponin-sensitivity (Fig 4C), which is consistent with the fact that ring-stage parasites do not display new permeability pathways and thus do not have high [Na^+^] within the host cell cytosol [9]. These results strongly support the notion that the influx of Na^+^ into the parasite cytosol is a requisite step preceding cholesterol accumulation in parasites treated with these drugs.

### Maduramicin, a Na^+^ ionophore, also induces cholesterol accumulation in trophozoites

**Fig 5:**
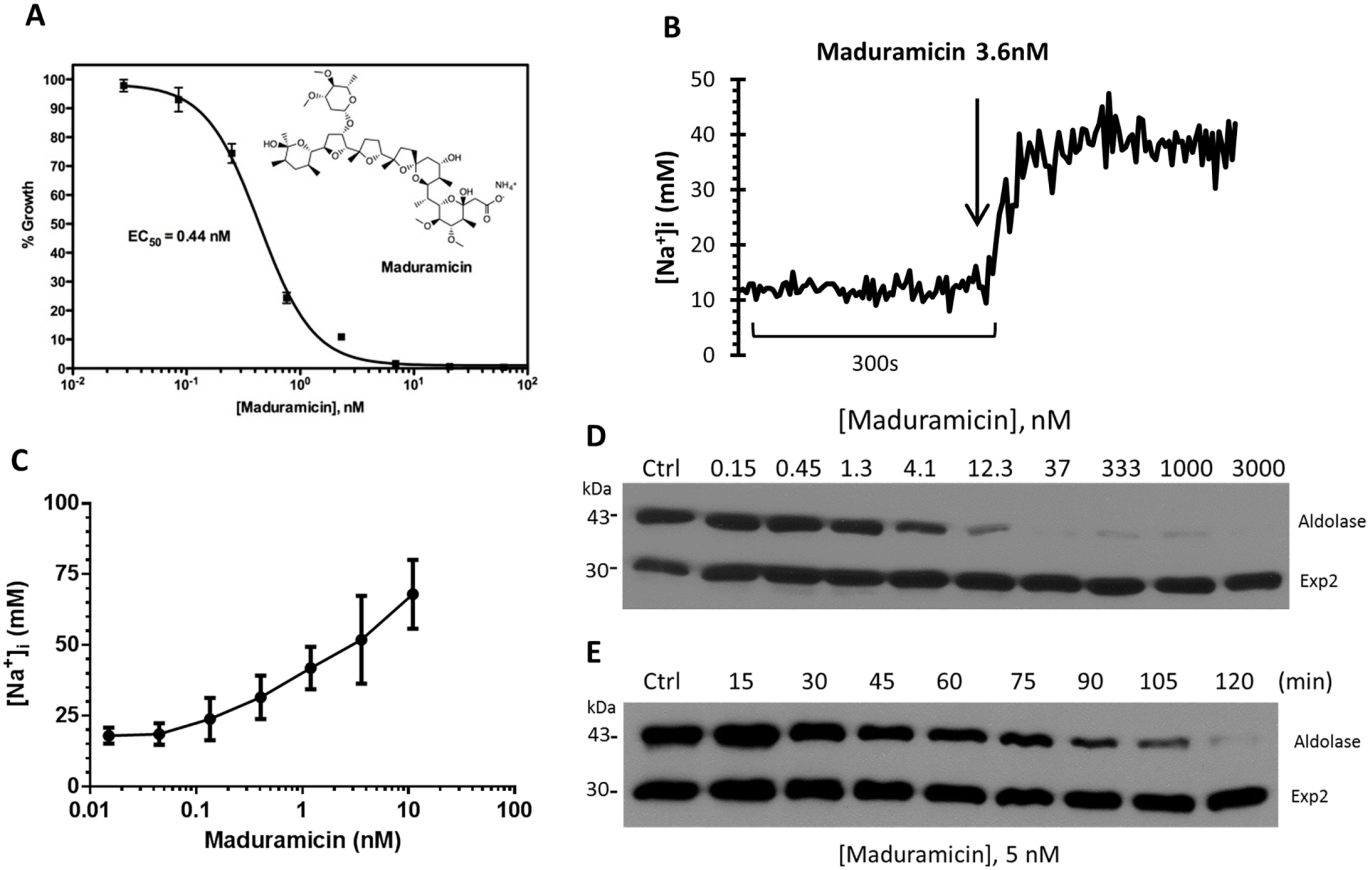
A monovalent ionophore, maduramicin, causes rapid Na^+^ influx and cholesterol incorporation into the trophozoite stage *P*. *falciparum*. (A) Growth inhibition by maduramicin was assessed by ^3^H-hypoxanthine incorporation by *P*. *falciparum* in a 48 h assay, with EC_50_ of 0.44 nM. (B) Maduramicin causes rapid influx of Na^+^ into *P*. *falciparum*. Ratiometric measurements of [Na^+^]_i_ were carried out as described in Materials and Methods. Addition of 3.6 nM maduramicin rapidly caused Na^+^ influx into the parasite. (C) The plateau levels of [Na^+^]_i_ at different concentrations of PA21A050 and maduramicin are shown, indicating a dynamic balance between Na^+^ influx and Na ^+^ efflux. (D) *P*. *falciparum* 3D7 trophozoites (30–34 h post-infection) were exposed to the vehicle (Ctrl) or the indicated doses of maduramicin for 2 h, followed by the assessment of saponin sensitivity as described in Fig 2. Leakage of cytosolic aldolase was assessed by SDS-PAGE and immunobloting. (E) *P*. *falciparum* 3D7 trophozoites were exposed to the vehicle (Ctrl) or 5 nM maduramicin for the indicated period of time. Treated parasites were released by mild saponin treatment and subjected to Western blot analysis using antibodies to aldolase or Exp2.

As reported previously, Na^+^ influx induced by treatment with KAE609 and PA21A050 is also accompanied by an increase in cytosolic pH within the parasites, presumably due to an inhibition of PfATP4 wherein Na^+^ pumping is believed to be countered by H^+^ import. Thus, the effects we describe here could potentially be due to an increase in cytosolic pH. We assessed this possibility by treating trophozoite stage parasites with maduramicin, a Na^+^ ionophore anti-coccidial drug (structure in the inset of Fig 5A), that has recently been shown to have potent antimalarial activity and synergy with PA21A050 [23]. As an ionophore, maduramicin is not likely to alter cytosolic pH, and thus effects of Na^+^ influx could be assessed independent of the pH change. As shown in Fig 5A, maduramicin inhibited P. falciparum growth with EC_50_ of 0. 44 nM. Within the range of concentrations used for parasite growth inhibition, no toxic effects or morphological changes were apparent in uninfected erythrocytes. Maduramicin induced rapid influx of Na^+^ into freed parasites (Fig 5B) at a very low concentration with half-maximal effect observed at 4 nM of the compound (Fig 5C). The initial rate of Na^+^ influx was much more rapid compared to that observed for KAE609 and PA21A050 (compare Fig 5B with S3 Fig). This is likely due to the ionophoric activity of maduramicin, which is expected to rapidly equilibrate [Na^+^] across a membrane, compared to the putative Na^+^ pump inhibitors. A 2 h treatment with maduramicin also induced saponin sensitivity to the PPM as judged by aldolase leakage with EC_50_ for this effect (~ 5 nM) being about 10-fold higher than the EC_50_ for growth inhibition (Fig 5D), as was observed for KAE609 and PA21A050. The time course of cholesterol accumulation within the parasites following the treatment with maduramicin was similar to that seen for compounds KAE609 and PA21A050 (Fig 5E; compare with Fig 2D and 2E). This suggests that the processes that are triggered by Na^+^ influx have similar lag period irrespective of the stimulus used for the influx.

### Cholesterol accumulation in trophozoites induced by the drugs is reversible

**Fig 6:**
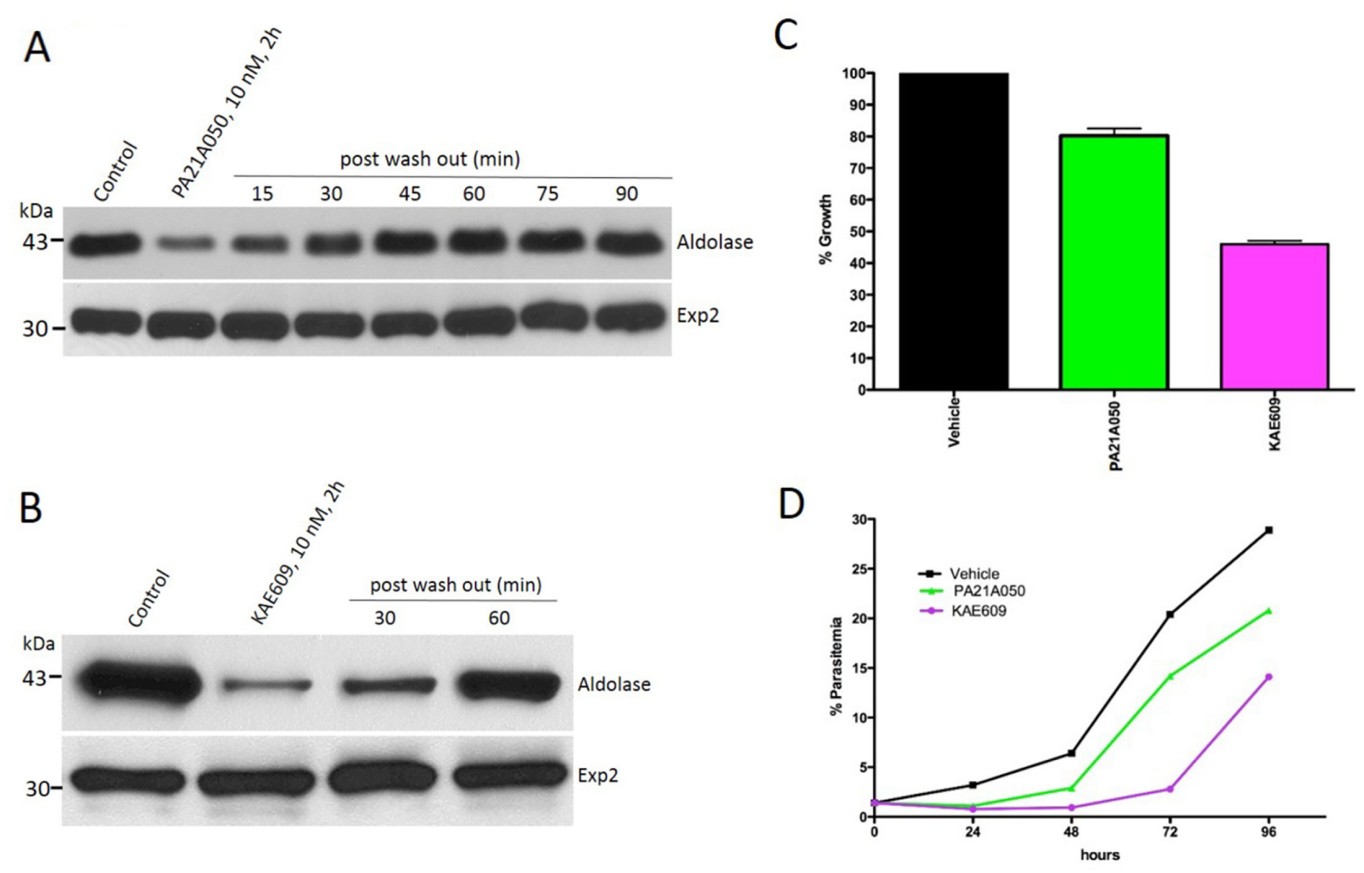
PA21A050 and KAE609 induced Cholesterol incorporation into the parasite is rapidly reversible. After 2 h of treatment, removal of PA21A050 (A) and KAE609 (B) rapidly leads to restoration of insensitivity to saponin-mediated aldolase loss within 30–60 min. Western blots were probed with antibodies to aldolase and Exp2. (C) After the treatment with PA21A050 (10 nM) or KAE609 (10 nM) for 2 h followed by the removal of compounds, ^3^H-hypoxanthine incorporation over a 24 h was assessed as a measure of parasite viability. (D) Parasite growth over a 96 h period following the 2 h compound treatment was assessed by measuring parasitemia in Giemsa stained smears every 24 h.

Malaria parasites do not synthesize cholesterol *de novo* [24]. In intraerythrocytic stages, the invading merozoite forms the PVM derived mostly from the RBC plasma membrane [16], and thus, the PVM contains a significant amount of host-derived cholesterol. As the parasite grows within the RBC, it ingests the host cytosol through structures called cytostomes, which incorporate portions of both the PVM and PPM in the process. Cholesterol and cholesteryl ester are detectable within the parasite [17,25] but the PPM remains poor in cholesterol despite its close interaction with the cholesterol-rich PVM, as indicated by the resistance to cholesterol-dependent detergents. Therefore, we wondered whether the parasites might have an active process to exclude cholesterol from the trophozoite stage. We examined parasites that were treated with the drugs for 2 h followed by removal of the drugs and assessed the saponin sensitivity of their PPM at various time points. Remarkably, within 60 min following the removal of either PA21A050 (Fig 6A) or KAE609 (Fig 6B) there was a complete reversal of saponin sensitivity. We interpret these results as suggesting that an active process of the parasite excludes cholesterol from the trophozoite stage, that this process is inhibited by the increased [Na^+^]_i_ caused by the drugs, and that the removal of the drugs following a 2 h exposure allows re-activation of the cholesterol exclusion process. PA21A050-treated trophozoite stage parasites showed a 20% reduction in ^3^H-hypoxanthine incorporation over a 24 h period following the 2 h exposure, whereas KAE609 treatment led to a 60% reduction (Fig 6C). Reduced parasite growth was also observed over a 96 h period following the drug exposure (Fig 6D).

### Na^+^ homeostasis disruption causes reversible coalescence of a GPI-anchored protein

**Fig 7:**
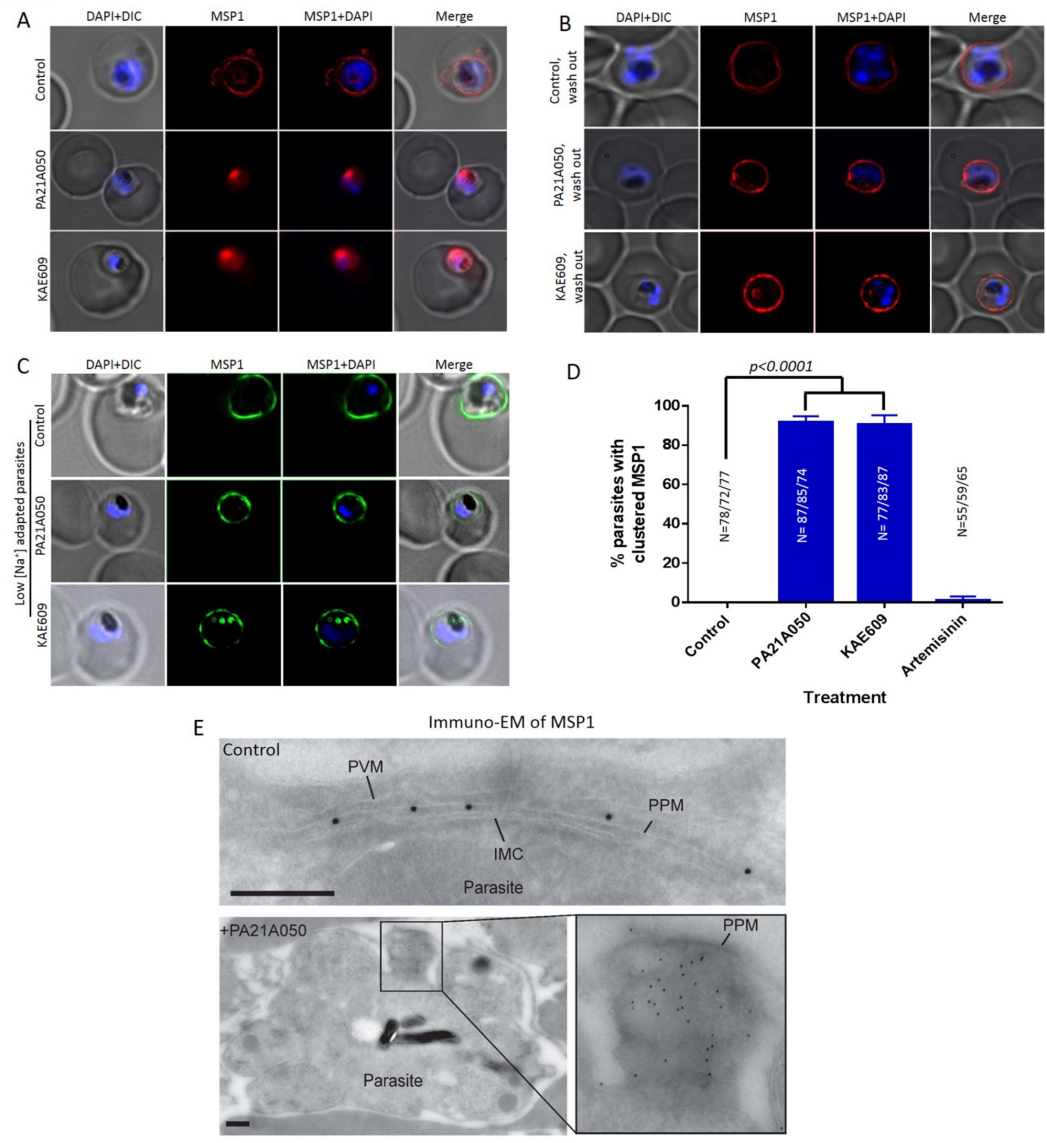
Reversible clustering of a GPI-anchored protein in compound treated trophozoite stage parasites. (A) Distribution of the GPI-anchored protein MSP1 was examined by immunofluorescence assays in 32–34 h PMI (post merozoite invasion) trophozoite stage *P*. *falciparum* 3D7 exposed for 2 h to the vehicle (control), PA21A050 (10 nM) or KAE609 (10 nM). (B) After 2 h of the removal of the compounds, parasites largely restored the distribution of MSP1 throughout the PPM. (C) The compound-induced MSP1 clustering was not observed in parasites adapted to grow in low [Na^+^] medium following compound treatments for 2 h. (D) Quantitation of parasites showing MSP1 clustering following treatment with the indicated compound for 2 h from 3 biological replicates (the total number of parasites (N) assessed is indicated above in each treatment condition). Error bars are the SD of the percentage of clustered MSP1 parasites determined under each experimental condition (N = 3). (E) Immunogold labeling for MSP1 on a representative *P*. *falciparum* control parasite (upper panel), in which the gold particles are distributed throughout the parasite plasma membrane (PPM); and on a representative PA21A050-treated parasite (lower panel), in which the gold particles are clustered in one patch. Cryo-sections were labeled with the anti-MSP1 antibody, and binding revealed with protein A-gold particles (10 nm). PPM, Parasite plasma membrane; PVM, Parasitophorous vacuole membrane; IMC, inner membrane complex. Scale bars are 100 nm. In each immunogold labeling experiment, 60–70 parasites were imaged, and Fig 6E shows representative images.

Cholesterol plays a critical role in membrane organization and formation of microdomains that display a higher concentration of signaling molecules including proteins embedded in the plasma membrane through GPI anchors [26–28]. Therefore, we next examined the effect of drug treatment on the localization of the merozoite surface protein-1 (MSP1), a GPI-anchored PPM protein [29,30]. MSP1 synthesis begins at the mid trophozoite stage and the protein is inserted into the PPM via a GPI anchor at its C-terminus. A 2 h exposure to 10 nM of either PA21A050 or KAE609 resulted in a dramatic clustering of MSP1 in >90% of the parasites (Fig 7A and 7D). Washing the drugs off from the parasites restored even distribution of MSP1 along the PPM in 2 h (Fig 7B). The MSP1 clustering was not observed when parasites adapted to grow in low [Na+] medium were exposed to PA21A050 or KAE609 (Fig 7C). Immunoelectron microscopy also revealed clustered MSP1 in PA21A050 treated parasites (Fig 7E). The clustering was also observed by IFA for another GPI-anchored protein, MSP-2 (S4 Fig). These observations suggest that rapid cholesterol accumulation in drug treated parasites may lead to clustering of the GPI-anchored proteins, which is reversible upon removal of the drugs and concurrent with restoration of a low cholesterol content in the parasite.

### Morphological changes following a short-term exposure to [Na^+^]_i_ disruptor drugs

**Fig 8:**
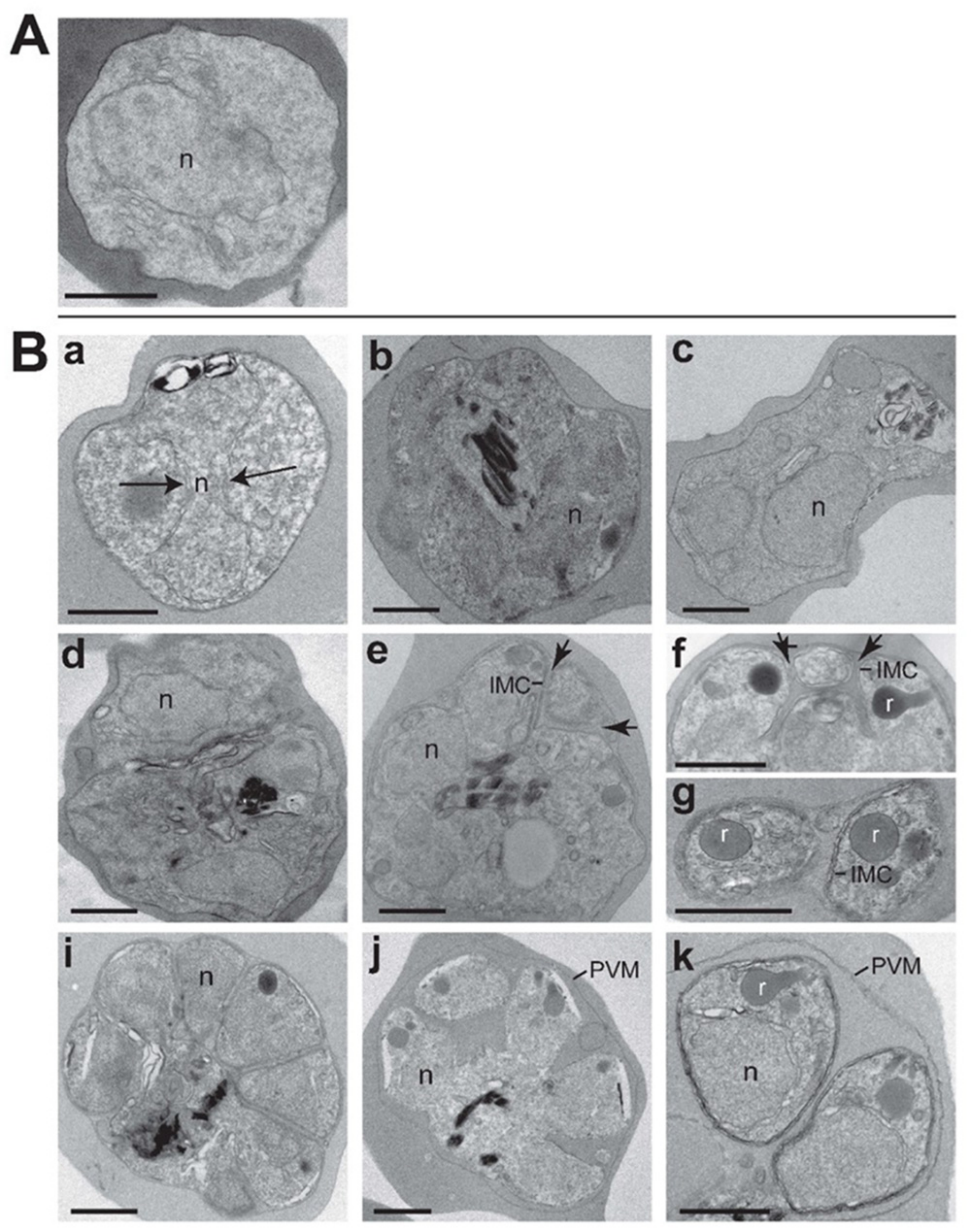
Ultrastructural representation of parasites exposed to compounds. Transmission electron micrographs of 32–34 h PMI (post merozoite invasion) trophozoite stage *P*. *falciparum* 3D7 infected erythrocytes. (A) untreated parasites and (B) parasites treated for 2 h with 10 nM KAE609 (panels a, c, e, f, i) or 10 nM PA21A050 (panels b, d, g, h, j) showing progressive steps in parasite transformation towards a schizogony-like stage characterized by scission of the nucleus (n; arrows in a), furrow cleavage demarked by the IMC (arrows in panels e, f) and secretory organelle biogenesis as illustrated for rhoptries (r). Scale bars are 250 nm.

Electron microscopy of drug-treated parasites revealed dramatic alterations in parasite morphology (Fig 8). Control parasites exposed to the vehicle were all uni-nucleated (Fig 8A; representative of images examined from 25 thin sections). In contrast, trophozoites exposed to KAE609 (Fig 8B, panels a, c, e, f, i) or PA21A050 (Fig 8B, panels b, d, g, h, j) for 2 h showed an acceleration in stage progression from trophozoites to aberrant schizont-like parasites. Images shown here are representative >75 thin sections examined for each drug treatment in two biological replicate experiment. Signs of schizogony included nuclei fragmentation (Fig 8B, panel a) resulting in accumulation of from two (Fig 8B, panels b, c) to six (Fig 8B, panels d, e) nuclear profiles, and initiation of cytokinesis, as evidenced by the wrapping of the inner membrane complex (IMC) around premature parasites that contain apparently mature rhoptries (Fig 8B, panels f, g). Several parasites exhibited more advanced stages of this atypical schizogony with the appearance of individualized parasites floating in the RBC cytoplasm and surrounded by a PVM like membrane (Fig 8B, panels h-j). Overall, 95% of the treated parasites showed at least one of the features associated with schizogony, with 70% of the parasites showing more than one nucleus in a given section.

**Fig 9:**
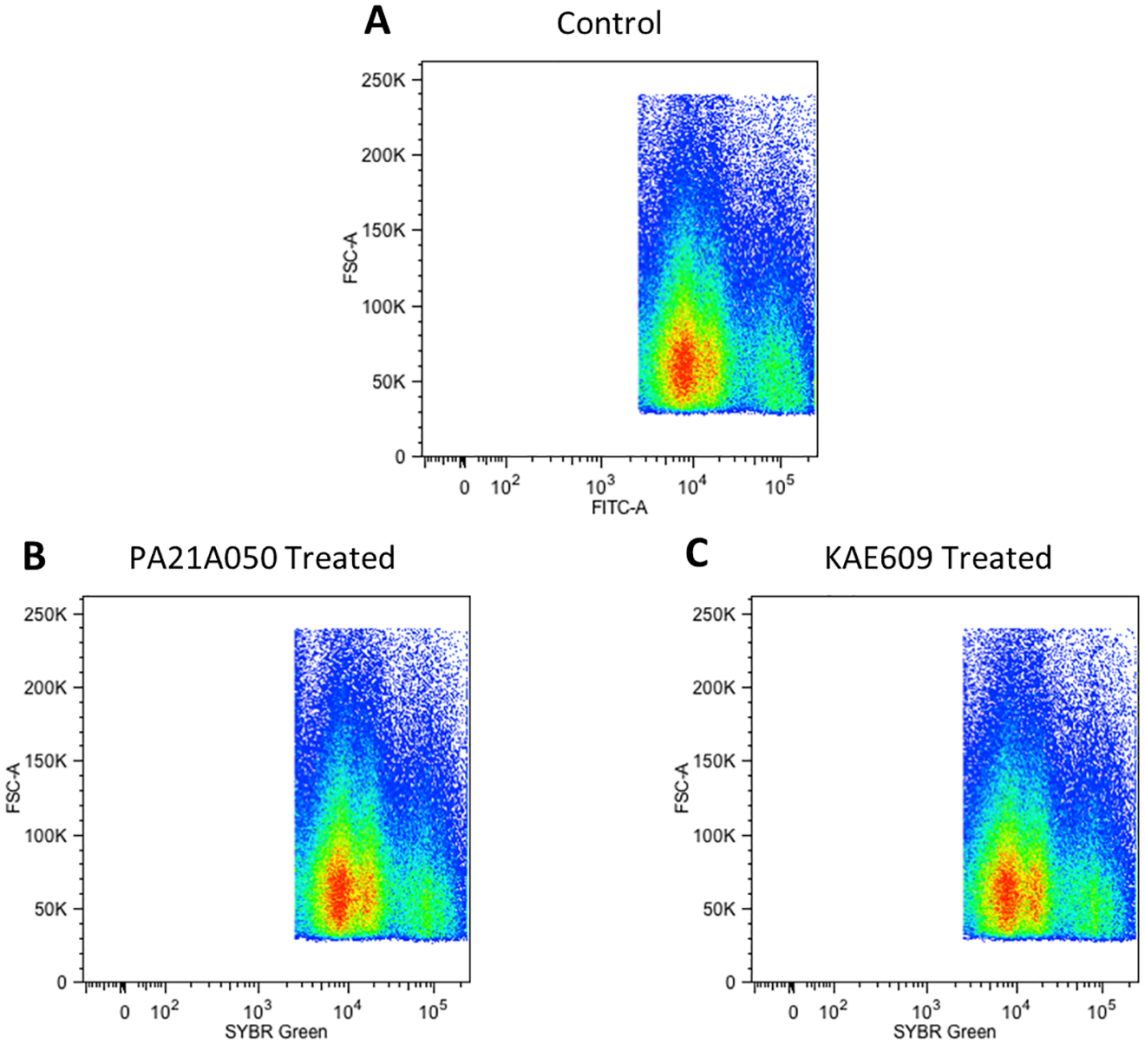
A 2 h treatment with PA21A050 or KAE609 did not show increased DNA content in *P*. *falciparum*. 32–34 h PMI (post merozoite invasion) trophozoite stage *P*. *falciparum* 3D7 parasites were exposed to the vehicle (Control) (A) or 10 nM PA21A050 (B) or 10 nM KAE609 (C) for 2 h, fixed and stained with SYBR Green to assess DNA content, as measured by fluorescence intensity detected using flow cytometry. Uninfected erythrocytes are gated out and pseudocolor density dot plots of the infected parasite populations are depicted.

We wondered whether the observation of multiple nuclei in some of the treated trophozoites indicated stimulation of DNA synthesis by these compounds. To assess this, the DNA content of treated parasites was examined by flow cytometry of SYBR Green stained parasites. As shown in Fig 9, the overall flow cytometry profiles of control and treated parasites did not change after a 2 h exposure to the drugs. Previous studies have shown that mid- to late stage trophozoites consist of sub-populations that have undergone DNA replication without nuclear division [31]. Thus, the acceleration of events normally associated with schizogony that are observed following the drug treatment does not appear to involve DNA replication per se, and the multiple nuclei seen in some of the treated parasites likely indicate an induction of nuclear division of polyploid nuclei present within various sub-populations of trophozoites [31].

## Discussion

The results above provide a glimpse of a complex set of events that are triggered by an influx of Na^+^ into the cytoplasm of trophozoite stages of *P*. *falciparum*. By using a simple saponin sensitivity assay, we have demonstrated profound changes in plasma membrane permeability of the trophozoite stage *P*. *falciparum* induced by two chemically distinct antimalarial drugs. We recognize that this approach does not directly measure the parasite plasma membrane properties. However, the membrane organization of intraerythrocytic parasites makes it technically very difficult, if not impossible, to separate PPM from PVM to carry out direct biochemical assessment of their content. Close proximity of the two membranes also makes it very difficult to use fluorescent probes to monitor their relative cholesterol contents. The approach of fluorescence lifetime imaging microscopy to assess cholesterol content of PPM vs. PVM does not provide adequate resolution in live imaging of parasites and thus is not suitable for this purpose [18]. Thus, we have relied on the well-documented propensity of saponin (and digitonin) to insert pores within membranes in a cholesterol-dependent manner, leading to the leakage of cytosolic proteins, to provide evidence for dramatic but reversible changes in the cholesterol content of the PPM induced by these antimalarial drugs. Experiments described in Fig 3, in which MβCD and MβCD loaded with cholesterol were used prior to saponin sensitivity assessment of treated and untreated freed parasites, provide strong support to our interpretations of rapid accumulation of cholesterol in the PPM by drug treated parasites.

Reasons for the very low cholesterol content of the PPM during intraerythrocytic development of the parasites have remained unexplored at this point. One possibility is that the low cholesterol content of the PPM reduces its rigidity, thus permitting greater flexibility to the intraerythrocytic parasite as it traverses through the host’s circulation. Uninfected erythrocytes in circulation need to endure a high level of shear stress as they pass through the tight spaces of the peripheral vasculature, and the high degree of deformability afforded by their plasma membrane-associated cytoskeleton permits erythrocytes to withstand this stress [32,33]. Parasite-infected erythrocytes also face the same shear stress that too needs to be mitigated in some manner [34,35]. Cytoadherence of infected erythrocytes bearing later stages of *P*. *falciparum* to endothelial cells is one mechanism that minimizes exposure to the stress [36,37]. Here, we propose that the reduced cholesterol content of the PPM resulting in reduced rigidity is another way by which the parasite manages to withstand the shear stress of circulation.

The results described here could also provide an explanation for the observation that, while the full lethal effects of the [Na^+^]_i_ disruptor drugs on *P*. *falciparum* culture appear to take 24 to 48 h (as judged by *in vitro* parasite clearance experiments [38]; F. J. Gamo, personal communication), parasites are cleared much more rapidly *in vivo*. Treatment with these drugs of immunodeficient mice, engrafted with human erythrocytes and infected with *P*. *falciparum*, cleared the parasites from circulation with a time course that was faster than any other antimalarials tested [5,6]. A clinical trial of KAE609 revealed that a single 30 mg dose resulted in clearance of both *P*. *falciparum* and *P*. *vivax* from the circulation of malaria patients to undetectable levels within 12 h [39], an unprecedented finding that has raised enthusiasm for this drug (and possibly others in its class) as a new weapon against malaria [2,40–42]. We suggest that the accumulation of cholesterol into the PPM induced by KAE609, PA21A050 and other [Na^+^]_i_ disruptor drugs (in addition to the morphological changes observed following drug exposure) results in increased rigidity of the membrane, rendering the parasite vulnerable to damage and/or removal from the circulation. This proposition is supported by a recent report showing increased rigidity of KAE609-treated parasitized erythrocytes when assessed in a microfluidic device *in vitro* [43].

In addition to imparting saponin sensitivity to the parasite, the [Na^+^]_i_ disruptor drugs also caused coalescence of the GPI-anchored plasma membrane proteins MSP1 and MSP2. Cholesterol, in conjunction with other lipids within the membrane, is involved in appropriate formation of microdomains wherein GPI-anchored proteins are present in greater concentration [28]. Although MSP1 is synthesized during the trophozoite stage and is displayed within the PPM, its function is critical at the surface of merozoites released at the end of the intraerythrocytic development cycle, where it has been shown to form a complex with other merozoite surface proteins, playing an essential role in host cell recognition and invasion by merozoites [44–46]. Recently, MSP1 processing was shown to result in activation of its spectrin binding function and aid in parasite egress from the RBC [47]; however, drug treatment of trophozoites for 2 h, while causing clustering of MSP1, did not result in its processing (S5 Fig). The clustering of MSP1 observed here following the drug treatments likely results from the increased cholesterol within the PPM and the absence of other merozoite surface proteins required for appropriate display of the complex. It is interesting to note that, unlike the PPM of the trophozoite stage parasites, merozoites have been reported to contain a substantial amount of cholesterol [48], which would be consistent with the suggestion that cholesterol is required for appropriate formation of the MSP1 containing complex involved in receptor interaction and invasion.

A remarkable observation was the reversibility of cholesterol accumulation and MSP1 clustering within minutes after the [Na^+^]_i_ disruptor drugs were washed away from the trophozoites. We interpret this to indicate the presence of an active process that excludes cholesterol from the parasite plasma membrane. We propose this putative “cholesterol pump” (in a manner similar to the Niemann-Pick disease associated protein NPC-1, which transports cholesterol from the endosomal compartment in mammalian cells [49]) to be present in the PPM and being responsible for maintaining low cholesterol levels, thereby preventing increased rigidity of the PPM. The net advantage to the parasite would be protection against damage and removal when passing through the narrow labyrinths of the peripheral vasculature. At the time of merozoite formation, however, this process of cholesterol exclusion would need to be reversed so that the merozoite plasma membrane could acquire cholesterol necessary for the appropriate display of merozoite surface proteins. Furthermore, cholesterol in the merozoite membrane is likely to be required for its rigidity and for imparting proper shape to this invasive extracellular stage. We hypothesize that the [Na^+^]_i_ disruptor drugs are able to reverse or inhibit this putative cholesterol pump.

Although most of the parasites appear to undergo rapid reversal of cholesterol accumulation and MSP1 clustering upon the removal of the drugs following a 2 h treatment, viability of the parasites appeared to be reduced to varying degrees. Ultrastructural examination of treated parasites provides a possible explanation for this variation. The drug treatment appears to trigger events resembling schizogony in the nominally trophozoite stage parasites. There appeared to be significant variations among parasites with regard to the morphological changes that were apparent by electron microscopy (Fig 8). As indicated by flow cytometry of DNA content (Fig 9), the trophozoite stage parasites used in these studies contained a significant sub-population that had higher DNA content than the majority, indicating that these parasites had undergone DNA replication and possessed polyploid nuclei. A potential explanation for the variation in viability could be that the parasites with polyploid nuclei [31] might not be able to recover from the premature schizogony-like events (nuclear division, formation of inner-membrane complex and rhoptries etc.) induced by the drugs.

**Fig 10:**
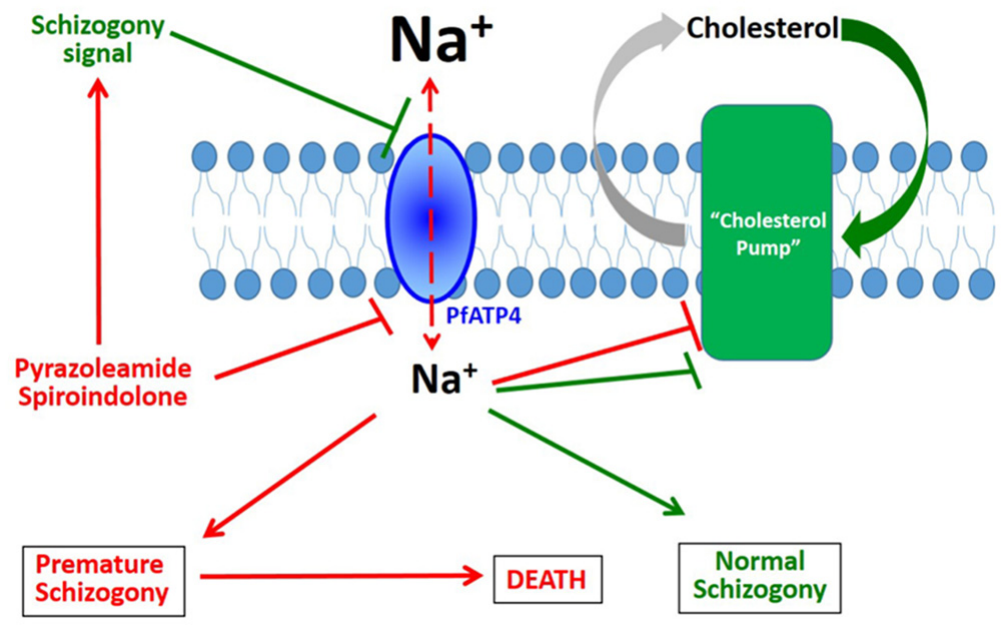
A schematic model showing Na^+^ dependent events in normal and drug-treated parasites. Green arrows and hammers indicate the proposed normal physiological process whereas red arrows and hammers indicate events triggered by treatment with [Na^+^]_i_ disruptor compounds. Under physiological conditions, a signaling event at the initiation of schizogony is hypothesized to inhibit Na^+^ pumping by the PPM-localized PfATP4, resulting in Na^+^ influx within the parasite cytoplasm. The increased [Na^+^]_i_ provides further signaling that leads to the inhibition of a putative cholesterol pump, resulting in increased accumulation of cholesterol in the PPM; the increased cholesterol, in turn, is required for the appropriate display of merozoite surface proteins. The increased [Na^+^]_i_ also constitutes a signal for further progression of normal schizogony processes such as nuclear division and formation of the inner membrane complex. Spiroindolones and pyrazoleamides prematurely usurp this process by either directly inhibiting PfATP4 or by mimicking a schizogony signal, either of which would result in influx of Na^+^ into the parasite cytoplasm. Premature inhibition of the putative cholesterol pump and induction of schizogony processes by high [Na^+^] would result in parasite death.

The observation that cholesterol accumulation and MSP1 coalescence in the PPM were not seen in treated parasites adapted to grow in low [Na^+^] medium constitutes the main evidence to support the conclusion that the massive changes induced by a short term exposure to the drugs are consequences of increased [Na^+^]_i_ within the parasite. Experiments with the Na^+^ ionophore maduramicin (Fig 5) further support this conclusion. Furthermore, these changes are not observed in ring-stage parasites even when grown in regular medium (Fig 4C). This is likely due to the fact that, in the absence of parasite-induced new permeability pathways, the host cell cytosol of the ring-stage parasites has yet to acquire high level of [Na^+^], thus precluding the influx of excess Na^+^ into the parasite cytoplasm [9]. Influx of Na^+^ beyond a threshold level, then, could be a normal physiological signal for initiation of a complex set of events resulting in schizogony and merozoite formation. We hypothesize that an inappropriate Na^+^ influx induced by the new antimalarial drugs investigated here leads to parasite demise. Fig 10 provides a schematic to depicts this hypothesis. Green arrows and hammers indicate a normal process of parasite development. Under normal physiological conditions, developmental signaling event at the initiation of schizogony is hypothesized as being able to inhibit Na^+^ pumping by the PPM-localized PfATP4, resulting in Na^+^ influx within the parasite cytoplasm. The increased [Na^+^]_i_ provides further signaling that leads to the inhibition of a putative cholesterol pump, resulting in increased accumulation of cholesterol in the PPM required for appropriate display of merozoite surface proteins as well as for imparting rigidity to the membrane and its shape. We also hypothesize that the increased [Na^+^]_i_ also constitutes a signal for further developmental progression such as nuclear division and formation of the inner membrane complex. Red arrows and hammers in Fig 10 indicate the processes hypothesized as being induced by the drugs. In this model, spiroindolones and pyrazoleamides prematurely usurp this finely tuned process by either directly inhibiting PfATP4 or by mimicking a developmental signal, either of which would result in influx of Na^+^ into the parasite cytoplasm. Premature inhibition of the putative cholesterol pump and induction of developmental progression, we hypothesize, would result in parasite death. This model predicts the existence of a complex cascade of events that are unleashed by Na^+^ influx. It would be of great interest to identify various players that may participate in this cascade.

## Materials and Methods

### Parasites

*P*. *falciparum* lines 3D7, Dd2, and Dd2-R21 were cultured using type O^+^ human RBC (purchased from Interstate Blood Bank, TN), in RPMI1640 supplemented with 15 mM HEPES, 2 g/L sodium bicarbonate, 10 mg/L hypoxanthine, 50 mg/L Gentamysin sulfate, and 0.5% Albumax (cRPMI). Asexual stages of *P*. *falciparum* were maintained at 5% haematocrit in cRPMI at 37°C in a humidified incubator containing 90% N_2_, 5% O_2_ and 5% CO_2_. Parasite cultures were periodically tested for Mycoplasma contamination to ensure that they were free of Mycoplasma.

### Antibodies

*Plasmodium* aldolase antibody was purchased from Abcam (cat no. ab38905). Anti-hemagglutinin (HA) antibody was purchased from Santa Cruz Biotechnology (cat. no. sc-393579). Antibodies to MSP1_42_ and Exp2 were raised in rabbits as described previously [50,51]. Rabbit anti-serum to MSP2 was obtained from MR4 (Malaria Research and Reference Reagent Resource Center).

### Treatment of *P*. *falciparum* with pyrazoleamide and spiroindolone

*P*. *falciparum* 3D7 and/or Dd2 ATP4-HA, Dd2-R21 parasites were grown up to 5% parasitemia in cRPMI. Cultures were synchronized three times using 500 mM alanine in 10 mM HEPES, pH 7.4 [52]. Synchronized trophozoite stage parasites were treated with the indicated concentration of the compounds or DMSO. For most experiments the treatment was carried out for 2 h. For some of the experiments, the time of treatment was varied as described in figure legends. To assess reversibility of compound effect, the treated parasites were washed with cRPMI and incubated with compound-free medium for the indicated length of time. The treated and rescued parasites were processed according to the experimental design as described below.

### Freeing trophozoite stage parasites with saponin, anthrolysin O or by hypotonic lysis

Synchronized trophozoite stage parasites were exposed to varying compound concentrations in cRPMI for 2 h, collected by centrifugation and resuspended in 5 volumes of cRPMI. Equal volumes of 0.04% saponin (in cRPMI) was added to achieve final concentration 0.02%. After quick inversions (4–5 times) parasites were collected by centrifugation and washed with PBS twice at 1500x g for 5 min.

Anthrolysin O [10] (a kind gift from Dr. Richard Rest, Drexel University) was activated with 5 mM dithiothreitol on ice for 30 min. Parasites were collected by centrifugation at 500x g for 5min and resuspended to 10% hematocrit in RPMI without Albumax to remove any remaining cholesterol from the culture medium. Anthrolysin O was added at final concentration of 3.2 μg/ml and incubated for 5 min at 37°C, followed by centrifugation at 1500x g for 5 min. The parasite pellet was resuspended in 20 x volumes of RPMI without Albumax. A second exposure to anthrolysin O at 8 μg/ml for 5 min at 37°C was carried out to ensure complete lysis of uninfected erythrocytes. Freed parasites were collected by centrifugation and washed with cRPMI to quench any remaining anthrolysin O with cholesterol present in Albumax.

For hypotonic lysis, compound treated trophozoite stage parasites were collected by centrifugation at 500x g for 5min and resuspended in hypotonic solution (2 mM NaCl, 10 mM Tric-Cl, pH 7.4) and incubated for 5 min at 37°C. After the incubation, parasites were collected by centrifugation and washed twice with PBS and finally collected at 1500x g for 5 min. This was followed by SDS-PAGE and imuunobloting (S2 Fig).

In another set of experiments shown in S2A Fig, hypotonically released untreated parasites were exposed to 5 mM MβCD in IRPMI at 37°C for 30 min. MβCD treated parasites were washed twice with cRPMI. Hypotonically released and MβCD treated parasites both were subjected to saponin treatment and harvested by centrifugation. Parasites from different treatment regimens were subjected for SDS-PAGE and immunobloting.

### Western blotting

Freed parasite pellets were lysed in RIPA buffer (20 mM Tris HCl (pH 7.5), 150 mM NaCl, 1 mM Na_2_ EDTA, 1 mM EGTA, 1% NP-40, 1% Na-deoxycholate, 2.5 mM Na-pyrophosphate, 1 mM β-glycerophosphate) containing protease and phosphatase inhibitors (Sigma-Aldrich, Inc, St. Louis, MO, USA) by a brief sonication at 4°C. In some experiments, parasite pellets were lysed in SDS loading buffer directly. The lysates were centrifuged at 15,000x *g* at 4°C for 30 min and the supernatant was used for the immunoblots after protein estimation using the Bradford reagent (Sigma). Before loading, the protein was mixed with gel loading buffer (50 mM Tris HCl pH 6.8, 10 mM DTT/100 mM β-Mercaptoethanol, 2% SDS, 0.1% bromophenol blue, 10% glycerol) and heated at 90°C for 5 min. Samples were resolved on 12% SDS–PAGE and proteins were transferred to methanol-activated polyvinylidene fluoride (PVDF) membrane (Millipore) using anode buffer (25 mM Tris HCl pH 10.4, glycine, 10% Methanol) and wet transferred for 2 h at 4°C. Membranes were blocked with 5% non-fat skim milk powder in 1x PBS overnight and probed with specific antibodies. Primary antibody dilution was made in 1x PBS containing Tween-20 (0.2%) and incubated with the membrane for 3 h at RT on a rocker. Primary antibody binding was detected by appropriate secondary antibodies conjugated to horseradish peroxidase (Santa Cruz Biotechnology). Dilution of secondary antibody was made in 1x PBS Tween-20 (0.2%). After each incubation, the membrane was washed with 1x PBS-Tween-20 (0.2%) for 5 min at least 5–6 times. The immunoblots were developed using the SuperSignal West Pico chemiluminescent substrate (Thermo Scientific, USA).

### Immunofluoroscence assays (IFA)

IFA of *P*. *falciparum* 3D7 infected RBCs were performed in solution. Infected RBCs were centrifuged at 500*g* for 5 min, washed twice, and re-suspended in PBS. Cells were fixed using 4% formaldehyde/0.0075% glutarldehyde in PBS for 20 min at 4°C. All subsequent steps were carried out at room temperature (24–26°C). Cells were permeabilzed using 0.1% Triton-X 100 in PBS for 30 min and washed twice with PBS. 3% BSA was used for blocking. Antibodies to MSP1 were added at 1:1000 dilution in PBS containing 0.01% Triton X-100 and incubated for 3–4 hrs. Specificities of MSP1 and MSP2 antibodies have been shown by immunoblots (S4 and S5 Figs). RBCs were pelleted at 500x *g*, washed and treated with appropriate Alexa 488- or 594-conjugated secondary antibodies (Molecular Probes, invitrogen, USA) at 1:500 dilution for 2 hrs. After washing 3–4 times, cells were incubated for 5 min with DAPI (0.1 μg/ml). Cells were imaged using a Nikon ECLIPSE TiE inverted microscope. Acquired IFA images were processed by Image J software.

### Ultrastructural analyses

For electron microscopy, preparations of *P*. *falciparum* 3D7 infected erythrocytes (DMSO or compound treated) were fixed in 2.5% glutaraldehyde (Electron Microscopy Sciences; EMS, PA) in 0.1 M sodium cacodylate buffer (pH 7.4) for 1 h at room temperature, and then processed for thin-section transmission electron microscopy as previously described [53]. Thin sections were examined with a Philips CM120 Electron Microscope (Eindhoven, the Netherlands) under 80 kV. For immunogold staining, infected erythrocytes were first fixed in 4% paraformaldehyde (PFA from EMS) in 0.25 M HEPES (pH 7.4) for 1 h at room temperature, then in 8% PFA in the same buffer overnight at 4°C. Samples were infiltrated, frozen and sectioned as described [54]. The sections were immunolabeled with rabbit anti-MSP1 antibody at 1/75 dilution in PBS/1% fish skin gelatin, followed by incubation with anti-IgG antibodies and 10 nm protein A-gold particles before electron microscopy.

### Parasite growth inhibition and rescue

Parasite growth inhibition was assessed by a modified version of the method originally described by Desjardin et al. [55]. The method assessed parasite growth as reflected by incorporation of ^3^H-hypoxanthine by parasites. *P*. *falciparum* 3D7 parasites in culture were exposed to graded dilutions of test compounds for 48 h and incorporation of ^3^H-hypoxanthine into parasite nucleic acids during the last 24 h was determined by liquid scintillation spectroscopy.

To assess rescue of treated parasites, synchronized *P*. *falciparum* parasites at 1% parasitemia in 3% hematocrit culture were treated with 10 nM PA21A050 or 10 nM KAE609 for 2h. Parasites were harvested and washed 3 times with low hypoxanthine-containing medium (LHM) and resuspended to the original volume with LHM. 100 μl of this culture was mixed with 100 μl of LHM containing 4 μC/ml ^3^H –hypoxanthine (Perkin Elmer) and plated in 8 wells of a 96 well plate. ^3^H-hypoxanthine incorporation was assessed as above. The remaining culture was resuspended in cRPMI and the parasitemia was monitored after every 24 h by Giemsa staining of thin blood smears.

### Cholesterol extraction and donation using MβCD and MβCD-cholesterol and compound treatment of saponin freed parasites

Synchronized *P*. *falciparum* 3D7 infected erythrocytes at trophozoite stage were gently treated with 0.02% saponin in cRPMI as described above. The freed parasites were immediately harvested by centrifugation and washed once with RPMI without Albumax. An aliquot of saponin-freed parasites was first treated with MβCD (5 mM in RPMI without Albumax (IRPMI) at 37°C for 30 min) or MβCD loaded with cholesterol. MβCD was loaded with cholesterol at 1:5 MβCD:cholesterol ratio as described by Caliceti et al. [56] and used at final concentration of MβCD at 2.5 mM. Following the exposure to MβCD and MβCD-cholesterol, parasites were harvested by centrifugation and washed with cRPMI and resuspended in cRPMI containing the indicated amount of the compound. After 2 h of compound treatment, an aliquot of parasites was harvested and treated with 0.02% saponin in cRPMI followed by immediate harvesting by centrifugation. Another aliquot of freed parasite was first treated with the compounds for 2 h at 37°C, harvested by centrifugation and then extracted with MβCD and MβCD loaded with cholesterol as above. Parasites were collected by centrifugation, washed with cRPMI and then briefly treated with 0.02% saponin in cRPMI prior to SDS-PAGE and immunobloting.

In another set of experiment, untreated saponin-freed parasites were treated with 2.5 mM MβCD in IRPMI at 37°C for 30 min. Parasites were then harvested and washed twice with IRPMI. Parasites were exposed to MβCD-cholesterol at final concentrations of 0.625. 1.25 and 2.5 mM and incubated in the presence or absence of 10 nM PA21A050 or 10 nM KAE609 at 37°C for 2h. After the incubation, harvested parasites were subjected to saponin treatment followed by SDS-PAGE and immunobloting.

### Biochemical assays for cholesterol content

Highly synchronized trophozoite stage parasites at 15% parasitemia (0.9 ml packed cell volume) were gently freed by treatment with 0.2% saponin. Freed parasites were collected and resuspended in incomplete RPMI medium. Freed parasites were divided into 3 aliquots. One aliquot was left as freed parasites. A second aliquot was extracted with 5 mM MβCD in incomplete RPMI for 30 min at 37°C. The third aliquot was extracted with MβCD as above and then resuspended in cholesterol-saturated MβCD followed by a 30 min incubation at 37°C. Parasites were collected by centrifugation and their cholesterol content was determined by using the Amplex Red Cholesterol Assay kit (ThermoFisher; catalog no. A12216) using the protocol recommended by the manufacturer.

### Parasite growth in low [Na^+^] medium

*P*. *falciparum* parasites were adapted to grow in low [Na^+^] medium according to the method described by Pillai et al. [22]. Briefly, the medium contained all ingredients specified for RPMI1640 except that NaCl, NaHCO_3_, and Na_2_HPO_4_ were replaced by 64.8 mM KCl, 28.6 mM KHCO_3_, and 5.64 mM K_2_HPO_4_. In addition, 84.3 mM sucrose was included in the medium. Addition of 10% human serum to this medium was estimated to result in about a 7 mM Na^+^ concentration. Using this low [Na+] medium, *P*. *falciparum* parasites were adapted by growing them for 10–12 generations. These adapted parasites were then used for various experiments.

### Assessing cytosolic [Na^+^] in saponin freed parasites

Intracellular [Na^+^] was determined using methods described by Spillman et al. [3]. Briefly, saponin freed parasites were loaded with the ratiometric sodium sensitive probe SBFI-AM (5.5 μM) (Invitrogen) and 0.01% w/v Pluronic F-127 (Invitrogen) in suspension (at 2.5–3.5 x 10^8^ parasites/mL) for 30min at 37°C in bicarbonate-free RPMI supplemented with 20 mM glucose, 10 mg/L hypoxanthine, 25 mM HEPES and 50 mg/L gentamycin sulphate (pH 7.1). The probe-loaded parasites were washed twice (2,000x g, 30 s) and resuspended to a final parasite concentration of 1.0–1.5 x 10^8^/mL in a saline buffer (125 mM NaCl, 5 mM KCl, 1 mM MgCl_2_, 20 mM glucose, 25 mM HEPES, pH 7.1). SBFI loaded parasites were excited at 340 nm and 380 nm with emissions recorded at 500 nm in a Horiba Scientific FluoroMax 3 spectrofluorometer. Auto-fluorescence corrected 340/380 nm emission fluorescence ratio were related to [Na^+^]_i_ using an average from 3 independent calibration curves for SBFI. Calibration plots were generated for SBFI loaded parasites in solutions containing 0, 10, 25, 50, 75, 100, 130 mM Na^+^, made by mixing solutions of 80 mM Na^+^/K^+^ gluconate and 50 mM NaCl/KCl (1 mM MgCl_2_, 20 mM glucose, 25 mM HEPES, pH 7.1) and [Na^+^]_i_ was equilibrated using a combination of the ionophores nigericin (5 μM), gramicidin (2.5 μM) and monensin (5 μM).

### Assessing protein synthesis by compound treated parasites

Protein synthesis was assessed by labeling with ^35^S-methinonine/^35^S-cysteine over a 2 h period in the absence or the presence of 10x EC_50_ of PA21A050 (10 nM), KAE609 (10 nM), cycloheximide (2000 nM), or artemisinin (100 nM). Briefly, parasite culture at 10% parasitemia was washed with methionine/cysteine free RPMI1640 supplemented with 0.5% Albumax. Labeling was done in 1 ml cultures at 5% hematocrit in methionine/cysteine free RPMI1640 containing 125 μCi/ml of Easy Tag ^35^S Protein Labeling Mix (Perkin Elmer). After 1 h of incubation, 0.5 ml of regular cRPMI supplemented with 1 mg/ml of unlabeled methionine and cysteine plus the indicated amount of the compounds was added to the cultures, and incubation was continued for an additional 1 h. The cultures were divided into two 1.5 ml tubes, centrifuged, and washed twice with Albumax-free RPMI. Parasites were resuspended and treated with either 0.02% saponin or anthrolysin O as described above. Parasite pellets were resuspended in SDS-PAGE loading buffer, followed by SDS-PAGE and autoradiography.

### Flow cytometry to assess the DNA content of parasites

Trophozoite stage *P*. *falciparum* 3D7 parasites at 6–7% parasitemia were treated with the vehicle (DMSO), 10 nM PA21A050 or 10 nM KAE609 for 2 h. Parasites were fixed with formaldehyde/glutaraldehyde and stained with the DNA-specific fluorescent dye SYBR Green. Parasites were washed twice and resuspended in 1X PBS as a single cell suspension. For each treatment, 3 million events were counted by flow cytometry using a Becton Dickinson LSR Fortessa Cell Analyzer, and the data were analyzed by FlowJo single cell analysis software. The pseudocolor density dot plot was used to represent the data.

## Supporting Information

S1 Table Biochemical assay of cholesterol content in freed parasites. (DOCX)

S1 Fig **(A) ATP4-HA transgenic Dd2 parasites [5] were exposed to the indicated dose of PA21A050 for the indicated period of time followed by mild saponin treatment to release the parasites and subjected to Western blot analysis using antibodies to HA or Exp2.** (B) Trophozoites of pyrazoleamide resistant Dd2-R21 line were exposed to the indicated dose of artemisinin for the indicated period of time followed by mild saponin treatment to release the parasites and subjected to Western blot analysis using antibodies to aldolase or Exp2. (C) Trophozoite stage *P*. *falciparum* 3D7 were exposed to the vehicle (Control) or indicated dose of PA21A050 or KAE609 for 2 h followed by digitonin treatment to release the parasites and subjected to western blot analysis using antibodies to aldolase or Exp2.
(TIFF)

S2 Fig Hypotonic lysis of drug treated parasites do not display saponin sensitivity. (A) Western blot of untreated trophozoite stage parasites hypotonically released and treated with MβCD and then treated with saponin. (B) Parasites treated with 10 nM PA21A050, 10 nM KAE609 and 100 nM Artemisinin were released hypotonically or by saponin. Western blot was probed with aldolase and Exp2.
(TIFF)

S3 Fig Pyrazoleamide and spiroindolone induce rapid influx of Na^+^ in the cytosol of saponin freed parasites. Saponin freed SBFI-loaded 32–34 h PMI (post merozoite invasion) trophozoite stage *P*. *falciparum* 3D7 parasites were examined for parasite cytosolic [Na^+^] after the addition of 10x EC_50_ of PA21A050 (A), PA21A092 ((B), KAE609 (C), NITD246 (D), and artemisinin (E). Ratiometric measurements of [Na^+^]_i_ were carried out as described in Materials and Methods.
(TIFF)

S4 Fig **(A) Western blot showing time dependent expression of MSP2 using anti-MSP2 rabbit polyclonal antibody form MR4 (Malaria Research and Reference Reagent and Resource Center).** (B) Western blot of 30–32 h trophozoite stage parasites showing MSP2 expression after the treatment of PA21A050 in a time dependent manner for 2 h. (C) Immunofluoroscence assays of parasites treated for 2 h with of PA21A050 or KAE609 using anti-MSP2 antibody. (D) Quantitation of parasites showing MSP2 clustering following treatment with the indicated drug for 2 h from 2 biological replicates (the total number of parasites (N) assessed is indicated above in each treatment condition). Error bars are the SD of the percentage of clustered MSP2 parasites determined under each experimental condition (N = 2).
(TIFF)

S5 Fig **(A) Western blot showing processing of MSP1 using anti-MSP1**_**(42)**_ **rabbit polyclonal antibody.** Parasites were harvested at the indicated time post-infection, corresponding to progression from the trophozoite to schizont stage. (B) Western blot of 30–32 h trophozoite stage parasites showing MSP1 expression after the treatment of PA21A050 for indicated time. Little proteolytic processing was observed during the 2 h treatment with PA21A050.
(TIFF)
