# Supplementary material for: Na+ Influx Induced by New Antimalarials Causes Rapid Alterations in the Cholesterol Content and Morphology of Plasmodium falciparum
Source: PLoS Pathog. 2016 May 26;12(5):e1005647. doi: 10.1371/journal.ppat.1005647 (PMC4881962; doi:10.1371/journal.ppat.1005647)
Supplement: S1 Table — (DOCX) [file ppat.1005647.s001.docx]

**Supplementary Table 1**

| Sample | Cholesterol content; nmol/mg protein (± SD) |
| --- | --- |
| Freed parasites | 33 (± 0.41) |
| MβCD extracted freed parasites | 17 (± 0.56) |
| MβCD extracted freed parasites reconstituted with cholesterol-saturated MβCD | 54 (± 2.8) |

Highly synchronized trophozoite stage parasites at 15% parasitemia (0.9 ml packed cell volume) were gently freed by treatment with 0.2% saponin. Freed parasites were collected and resuspended in incomplete RPMI medium. Freed parasites were divided into 3 aliquots. One aliquot was left as freed parasites. A second aliquot was extracted with 5 mM MβCD in incomplete RPMI for 30 min at 37^o^ C. The third aliquot was extracted with MβCD as above and then resuspended in cholesterol-saturated MβCD followed by a 30 min incubation at 37 ^o^C. Parasites were collected by centrifugation and their cholesterol content was determined by using the Amplex Red Cholesterol Assay kit (ThermoFisher) using the protocol recommended by the manufacturer. Values given are average of 4 measurements ± standard deviation.
