# Supplementary figures and images for: Na+ Influx Induced by New Antimalarials Causes Rapid Alterations in the Cholesterol Content and Morphology of Plasmodium falciparum
Source: PLoS Pathog. 2016 May 26;12(5):e1005647. doi: 10.1371/journal.ppat.1005647 (PMC4881962; doi:10.1371/journal.ppat.1005647)

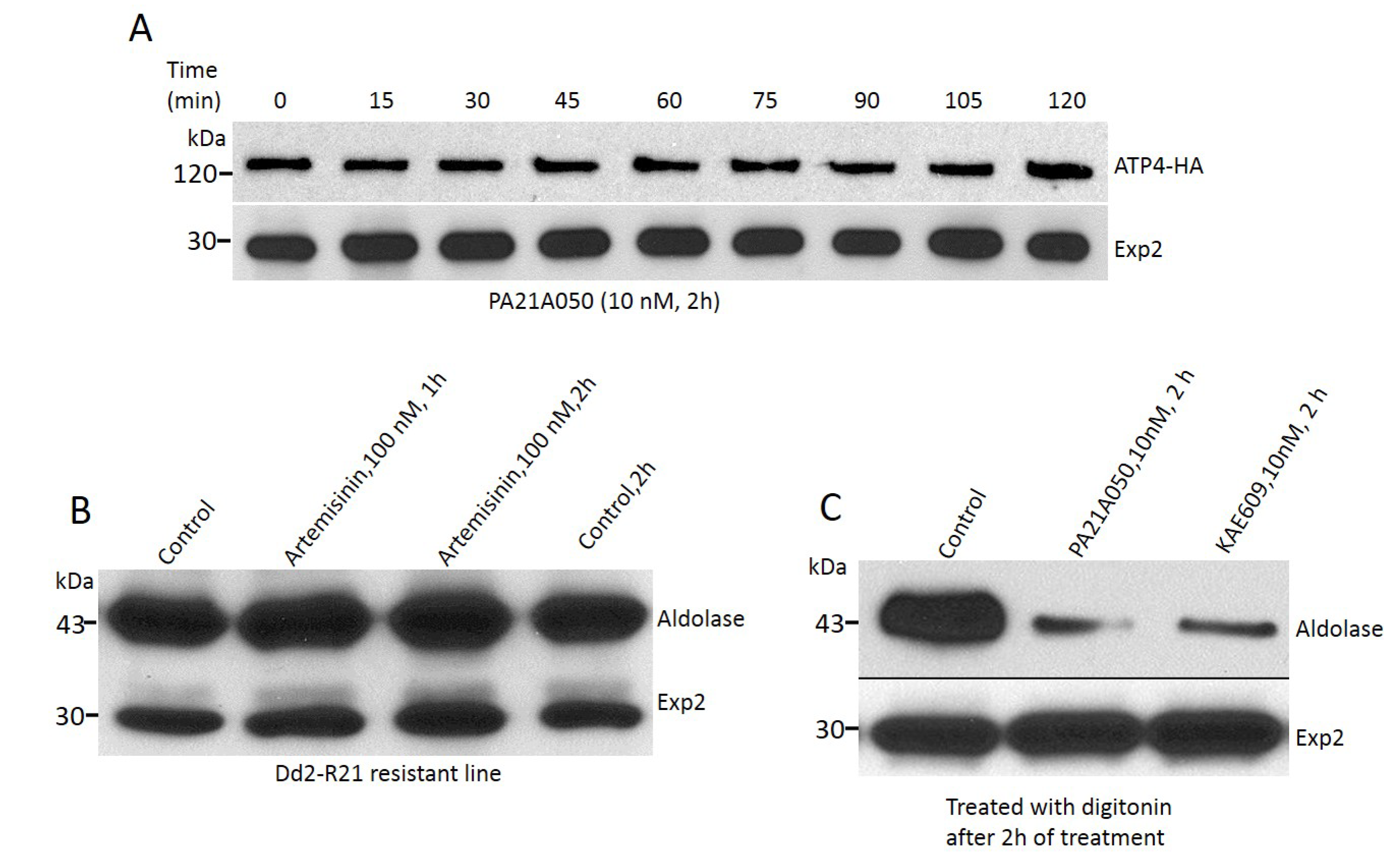

Supplement: S1 Fig — (A) ATP4-HA transgenic Dd2 parasites [5] were exposed to the indicated dose of PA21A050 for the indicated period of time followed by mild saponin treatment to release the parasites and subjected to Western blot analysis using antibodies to HA or Exp2. (B) Trophozoites of pyrazoleamide resistant Dd2-R21 line were exposed to the indicated dose of artemisinin for the indicated period of time followed by mild saponin treatment to release the parasites and subjected to Western blot analysis using antibodies to aldolase or Exp2. (C) Trophozoite stage P. falciparum 3D7 were exposed to the vehicle (Control) or indicated dose of PA21A050 or KAE609 for 2 h followed by digitonin treatment to release the parasites and subjected to western blot analysis using antibodies to aldolase or Exp2. (TIFF) [file ppat.1005647.s002.tiff]

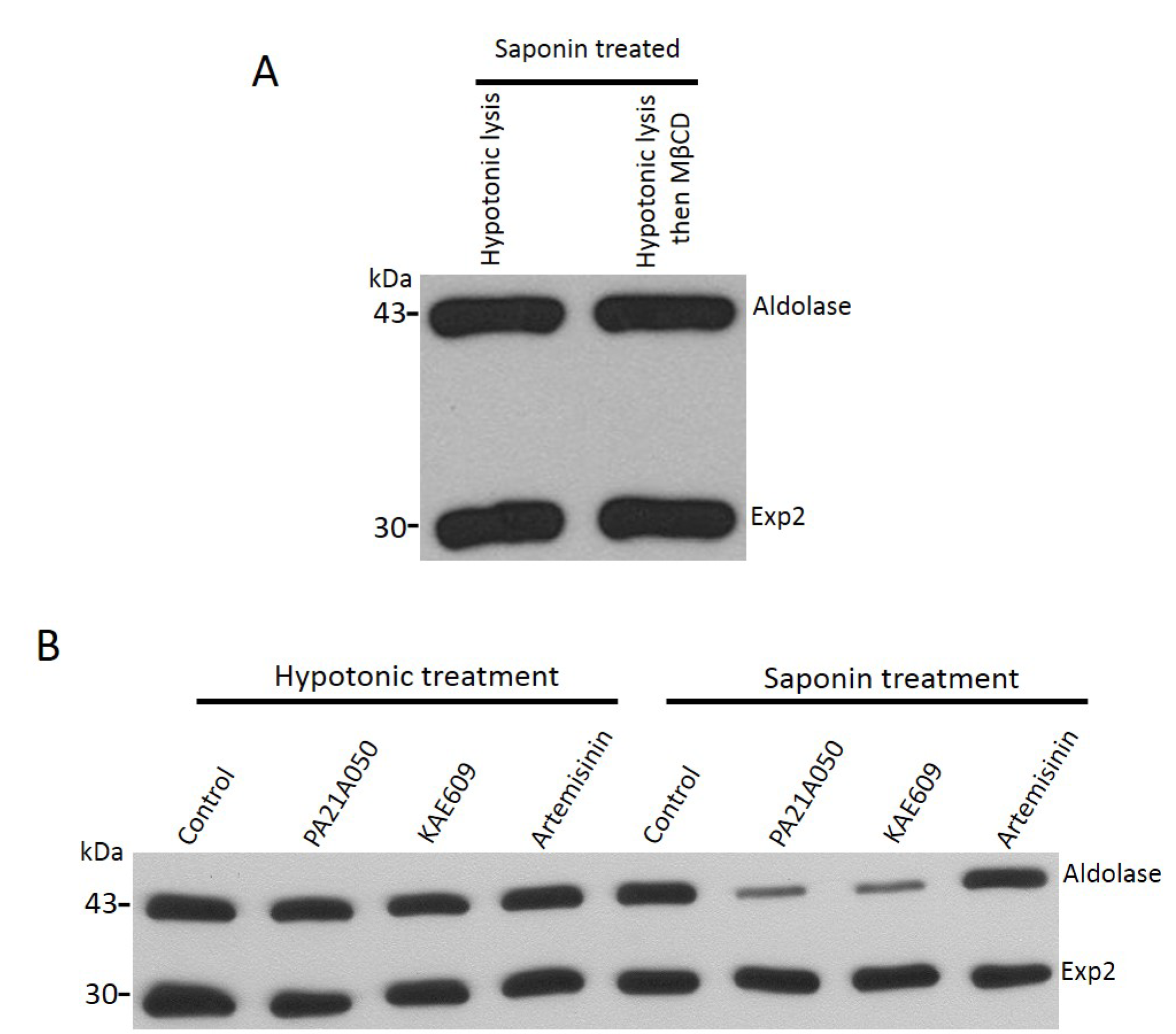

Supplement: S2 Fig — (A) Western blot of untreated trophozoite stage parasites hypotonically released and treated with MβCD and then treated with saponin. (B) Parasites treated with 10 nM PA21A050, 10 nM KAE609 and 100 nM Artemisinin were released hypotonically or by saponin. Western blot was probed with aldolase and Exp2. (TIFF) [file ppat.1005647.s003.tiff]

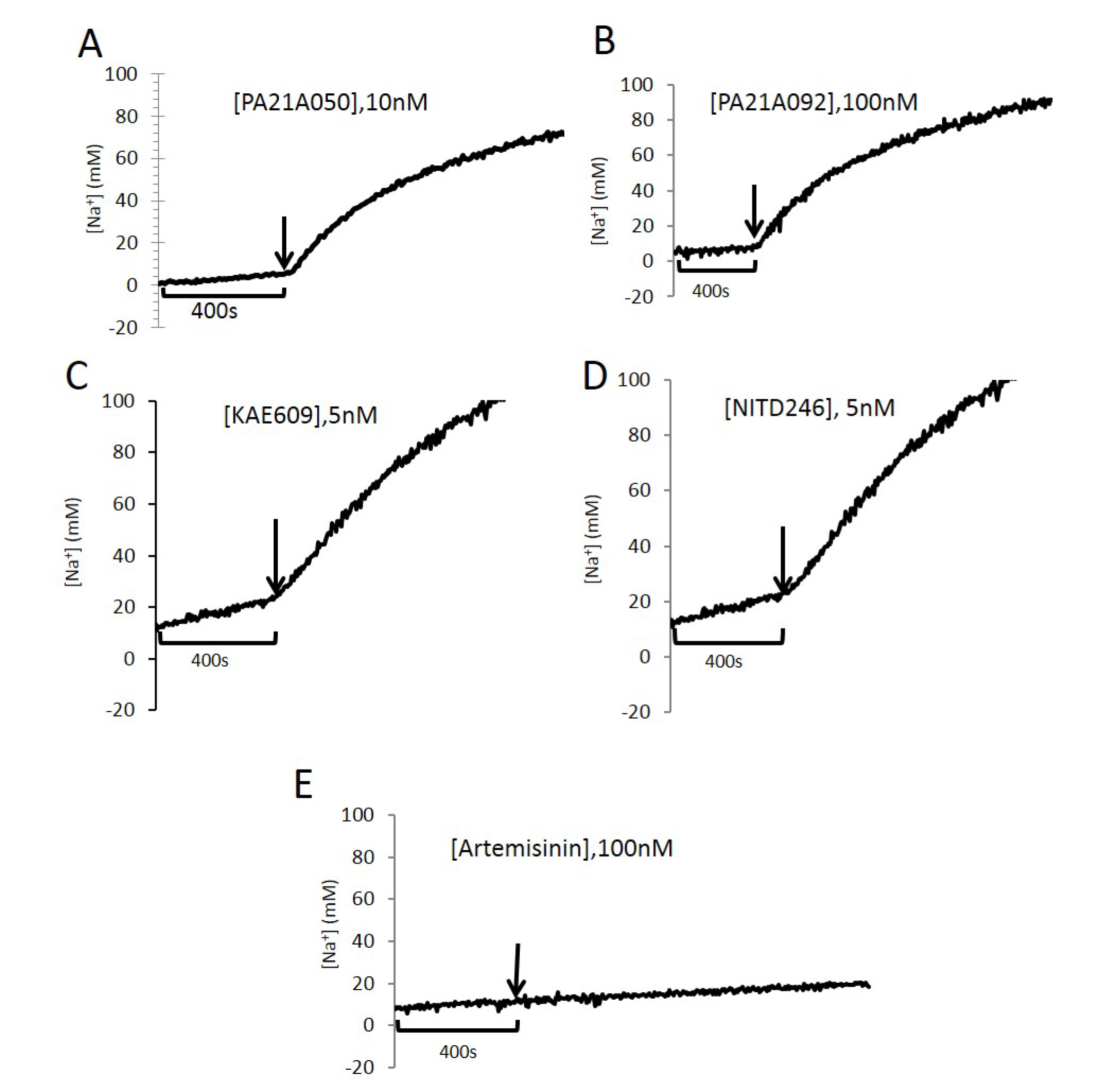

Supplement: S3 Fig — Saponin freed SBFI-loaded 32–34 h PMI (post merozoite invasion) trophozoite stage P. falciparum 3D7 parasites were examined for parasite cytosolic [Na+] after the addition of 10x EC50 of PA21A050 (A), PA21A092 ((B), KAE609 (C), NITD246 (D), and artemisinin (E). Ratiometric measurements of [Na+]i were carried out as described in Materials and Methods. (TIFF) [file ppat.1005647.s004.tiff]

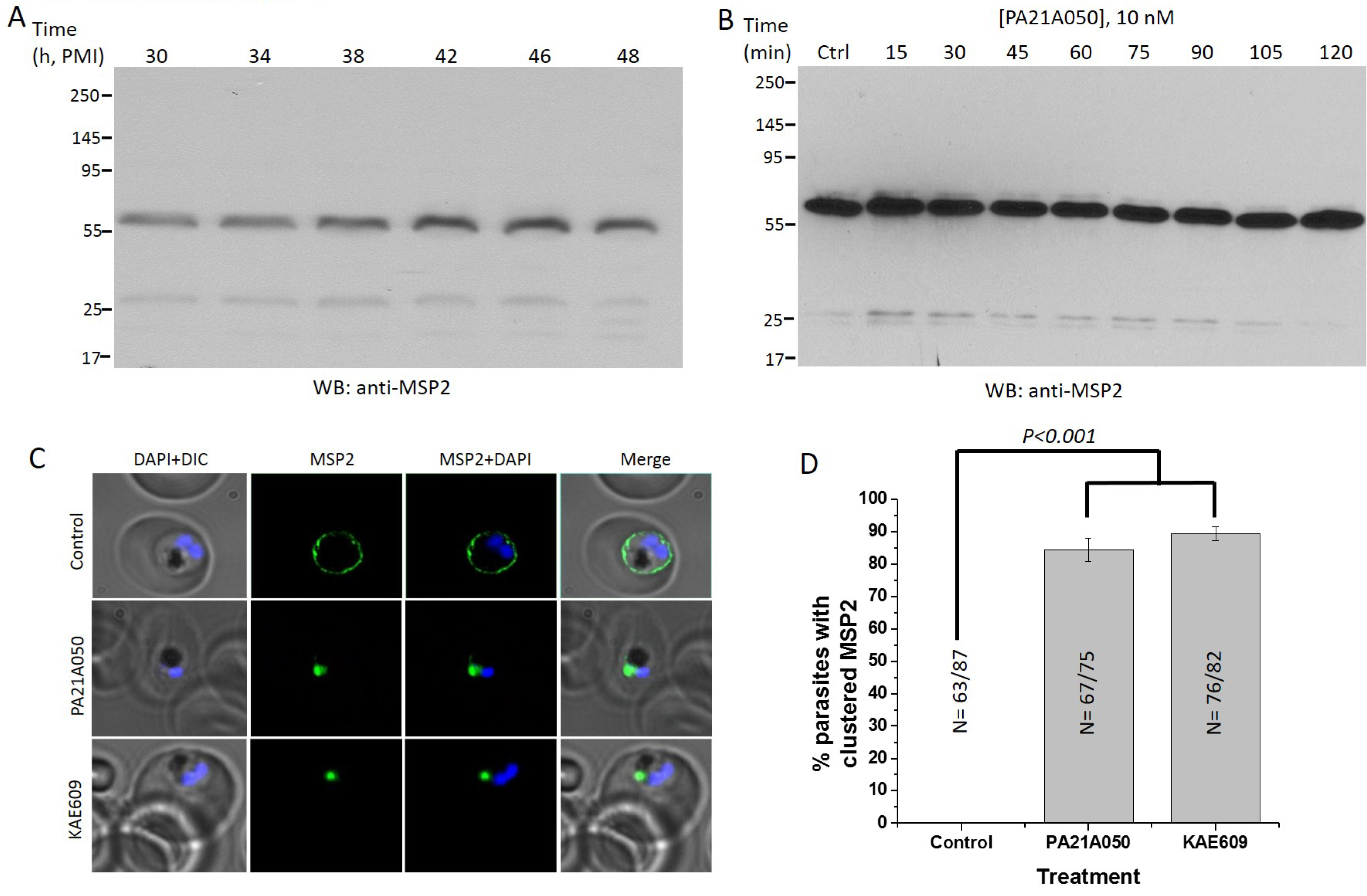

Supplement: S4 Fig — (A) Western blot showing time dependent expression of MSP2 using anti-MSP2 rabbit polyclonal antibody form MR4 (Malaria Research and Reference Reagent and Resource Center). (B) Western blot of 30–32 h trophozoite stage parasites showing MSP2 expression after the treatment of PA21A050 in a time dependent manner for 2 h. (C) Immunofluoroscence assays of parasites treated for 2 h with of PA21A050 or KAE609 using anti-MSP2 antibody. (D) Quantitation of parasites showing MSP2 clustering following treatment with the indicated drug for 2 h from 2 biological replicates (the total number of parasites (N) assessed is indicated above in each treatment condition). Error bars are the SD of the percentage of clustered MSP2 parasites determined under each experimental condition (N = 2). (TIFF) [file ppat.1005647.s005.tiff]

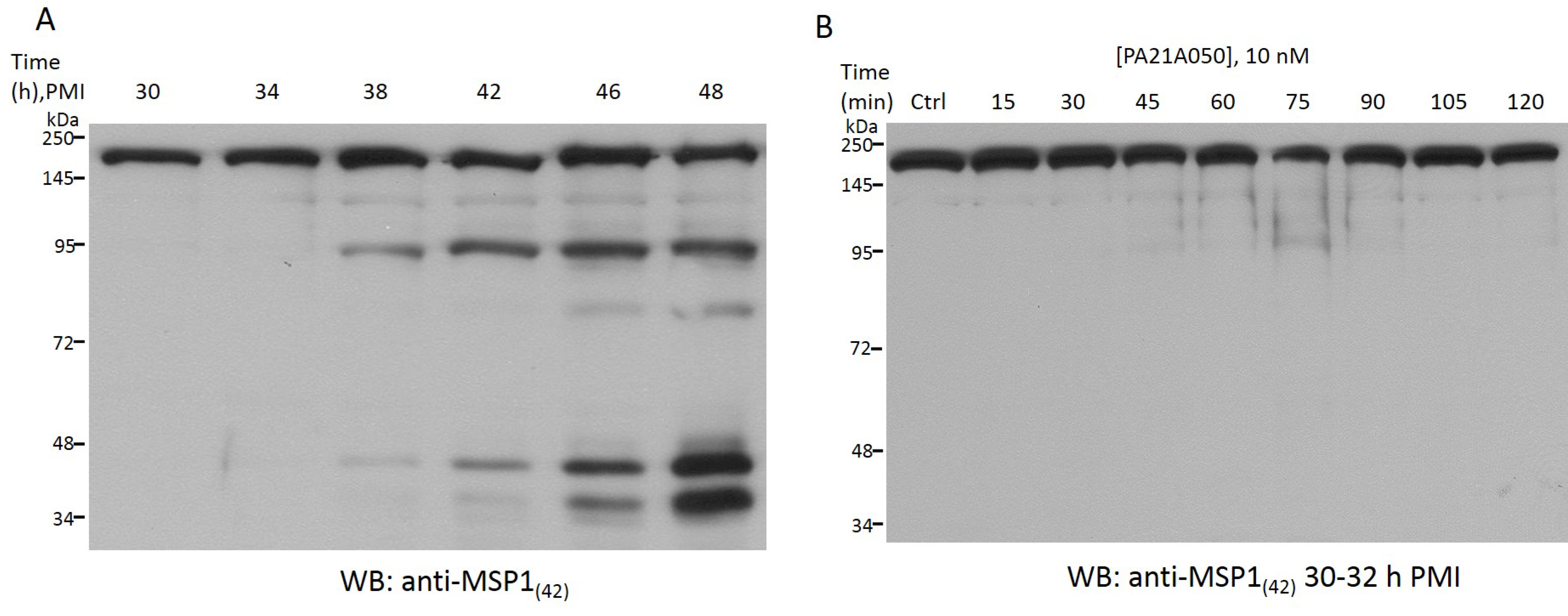

Supplement: S5 Fig — (A) Western blot showing processing of MSP1 using anti-MSP1 (42) rabbit polyclonal antibody. Parasites were harvested at the indicated time post-infection, corresponding to progression from the trophozoite to schizont stage. (B) Western blot of 30–32 h trophozoite stage parasites showing MSP1 expression after the treatment of PA21A050 for indicated time. Little proteolytic processing was observed during the 2 h treatment with PA21A050. (TIFF) [file ppat.1005647.s006.tiff]
